# *Acinetobacter* bacteria could be potent degraders of fragmented polyethylene and polypropylene among the digestive tract bacteria of *Galleria* waxworms

**DOI:** 10.1038/s41598-026-40931-7

**Published:** 2026-03-09

**Authors:** Takamasa Oota, Serisa Ebina, Hodaka Shimoura, Ying Huang, Kenji Miyamoto, Maki Teramoto

**Affiliations:** 1 Department of Marine Resource Science, Kochi University, 200 Otsu, Monobe, Nankoku, Kochi 783-8502 Japan; 2 Department of Biosciences and Informatics, Keio University, 3-14-1 Hiyoshi, Kohoku-ku, Yokohama, Kanagawa 223-8522 Japan

**Keywords:** *Acinetobacter*, *Galleria* waxworm, Polyethylene, Polypropylene, Digestive tract, Ghostshark, Biotechnology, Environmental sciences, Microbiology

## Abstract

Polyethylene (PE) and polypropylene (PP) dominate plastic production and are seriously contaminating environments. Bacteria in PE- and PP-ingesting worms, including *Galleria*, have thus attracted considerable attention owing to their PE and PP biodegradation potential and were investigated in this study. As PE and PP may be biodegraded after fragmented, the bacteria were enriched on *n*-hexadecane (a PE fragment) and on pristane (a branched alkane structurally similar to PP fragments) from the digestive tracts of *Galleria* waxworms. In these enriched cultures, *Acinetobacter* bacteria, particularly *Acinetobacter courvalinii*, *Acinetobacter calcoaceticus*, and *Acinetobacter pittii*, were detected most abundantly (*A. calcoaceticus* is closely related to *A. pittii*). Two alkane degraders probably of *A. courvalinii* and *A. pittii* were isolated from the cultures. They also degraded liquid PP (PP fragments), but not as a sole carbon source, and preferred different alkane chain lengths. On solid PE and PP, only initial signs of degradation (oxidation) were detected with each isolate. Surprisingly, the *Acinetobacter* bacteria were naturally abundant in the intestine of a ghostshark that contains much diacyl glyceryl ethers in its liver. These results indicate that *Acinetobacter* could be potent degraders of alkyl chains, including PE and PP fragments, among digestive tract bacteria of some terrestrial and marine organisms, including PE- and PP-ingesting worms.

**Supplementary Information:**

The online version contains supplementary material available at 10.1038/s41598-026-40931-7.

## Introduction

In 2019, 368 million tonnes of plastics were produced globally^1^. Polyethylene (PE) and polypropylene (PP) dominate plastic production^1^. These plastics are durable materials used in disposable products, and their unwanted release into the sea is causing serious problems. Uncontrolled plastics could be physically and chemically harmful to wildlife and humans^2,3^. Under these circumstances, bacteria that degrade PE and PP could remediate the plastic-contaminated environments.

The biodegradability of PE and PP is greatly promoted by abiotic factors, such as UV radiation, that cause chain cleavage (reduction of molecular mass) and oxidation^4–6^. Many bacteria degrade PE structures (*n*-alkanes, PE fragments)^7^. However, PE is biodegraded generally only with up to 10% weight loss in more than 2 months^8^, indicating that highly crystalline and long molecular structures limit biodegradation. Compared to PE, PP biodegradation has scarcely been reported. *Aneurinibacillus*^9^, *Bacillus*^10,11^, *Brevibacillus*^9^, *Gordonia*^12^, *Pseudomonas*^10^, *Rhodococcus*^11^, and *Streptomyces*^13^ bacteria have been suggested to degrade PP as evaluated by weight loss, morphological deterioration, oxidation modification, and growth on PP. *Aspergillus* fungi have also been indicated to degrade PP by growth on PP^4,5^. However, some of this weight loss could be attributed to biodeterioration where small fragments that separate from larger fragments could be failed to be measured. In contrast, PP structures (PP oligomers, liquid PP, PP fragments; branched alkanes) have been shown to be biodegraded for the first time by *Alcanivorax* bacteria that live in marine environments by monitoring liquid PP degradation using GC-MS: at least short PP oligomers have been completely degraded^14^. For final proof of biodegradation of the PP structure, such detection with GC-MS or detection of CO_2_ derived from PP can be considered. Although recently *Nitratireductor* and *Oricola* have also been shown to degrade PP structures using liquid PP and GC-MS^15^, no other genus has been reported to show clear biodegradability of PP structures. Branched alkanes appear more resistant to biodegradation than *n*-alkanes in natural fresh and sea water containing its indigenous microbes^16,17^ or by isolates, including *Alcanivorax* strains^18–21^. Anthropogenically occurring PP structures have more methyl branches than naturally occurring isoprenoid-derived branched alkanes and could be more resistant to biodegradation in natural fresh and sea water. Consistently, *Alcanivorax* bacteria do not use liquid PP as their sole carbon and energy source^14^. In contrast, *Nitratireductor* and *Oricola* have recently been shown to degrade liquid PP of all lengths detected, pristane, and *n*-hexadecane (C_16_) to a similar extent (moderately thermophilic PP degraders)^15^, indicating that the preference for less branched alkanes is not a general characteristic of branched-alkane-degrading bacteria. Some *n*-alkane degraders, including *Alcanivorax*, *Nitratireductor*, and *Oricola*, clearly degrade isoprenoid-derived branched alkanes (including pristane), but others do not^15,18–21^.

The biodegradation mechanism of *n*-alkanes is well-understood^22^, whereas that of branched alkanes is poorly understood. Biodegradation of isoprenoid-derived branched alkanes is indicated to involve the oxidation of the isopropyl terminus, followed by β-oxidation^18,23,24^. It is thus conceivable that PP can be degraded in a similar manner. Although genes for degrading isoprenoid-derived branched alkanes have not been identified, pristane induces the transcription of *n*-alkane hydroxylase genes (*alkB*, *p450*, and *almA*) in *Alcanivorax*^25,26^, suggesting that *n*-alkane hydroxylases may also be involved in the hydroxylation of the isopropyl terminus of branched alkanes.

PE- and PP-ingesting worms and their digestive tract bacteria have attracted considerable attention for their potential for biodegradation of PE and PP. The worms include waxworms of *Galleria mellonella* that ingest PE^27,28^ and PP^29,30^ and of *Plodia interpunctella* that ingest PE^31^ and yellow mealworms of *Tenebrio molitor* that ingest PE^32^. Waxworms of *G. mellonella* and *P. interpunctella*, as the name indicates, ingest beeswax^33^. Beeswax contains long *n*-alkanes and their derivatives^34^. Therefore, waxworms could ingest PE. *Galleria* waxworm saliva oxidizes PE (a sign of biodegradation)^35^. In addition, PE- or PP-oxidizing bacteria have been found from the digestive tract after culturing the indigenous bacteria in an inorganic medium containing PE or PP as the sole carbon source. Worms and bacteria could contribute synergistically to the biodegradation. The PE-oxidizing bacteria include *Enterobacter* from *Plodia*^31^ and *Galleria* waxworms^36^, *Bacillus* from *Plodia* waxworms^31^ and yellow mealworms^37^, and *Acinetobacter* from yellow mealworms^37^. PP-oxidizing bacteria include *Bacillus* from *Galleria* waxworms^30^. *Acinetobacter* and *Bacillus* from yellow mealworms in combination, but not alone, have grown on PE and reduced PE weight by 18% after 30 days^37^. However, *Acinetobacter*, *Bacillus*, and *Enterobacter* do not become abundant in the digestive tract of PE-fed *Galleria* waxworms^38,39^ and yellow mealworms^32^. Instead, *Serratia*^38^ or *Pseudomonas*^39^ have predominated in PE-fed *Galleria* waxworms. Although PE-degrading activity of the predominant *Serratia* and *Pseudomonas* bacteria has not been reported^38,39^, *n*-alkane-degrading activity has been reported in these genera^40^. These inconsistencies in the identified bacterial types could be due to the resistance of PE and PP to biodegradation, and the long molecular structures of PE and PP may be biodegraded after fragmented. Therefore, to identify the bacteria that can highly degrade the fragmented PE and PP, the bacteria were enriched on C_16_ (a PE fragment) and pristane (a branched alkane structurally similar to PP fragments) from the digestive tracts of *Galleria* waxworms in this study. In addition, we obtained a purple ghostshark of *Hydrolagus purpurescens* from the deep sea while searching for bacteria with health benefits, and ghostsharks characteristically contain much diacyl glyceryl ethers in their liver^41,42^. The bacterial community composition in the intestine and on the body surface of the ghostshark was surprisingly similar to the composition that was observed after the enrichment on C_16_ and pristane from the digestive tracts of the waxworms. These data were also shown in this study.

## Results and discussion

### Enrichment of *Acinetobacter* from waxworm digestive tracts on C_16_ and pristane

**Fig. 1:**
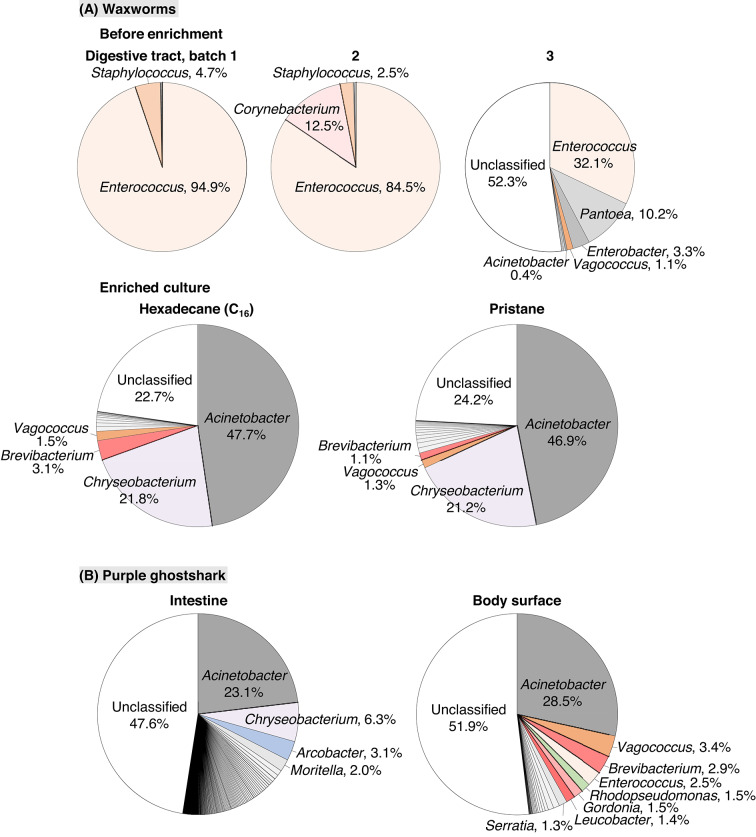
Bacterial community composition at the genus level in the digestive tracts of waxworms and the *n*-hexadecane (C_16_)- or pristane-enriched cultures (**A**) and in the intestine and on the body surface of a purple ghostshark (*Hydrolagus purpurescens*) (**B**). Data were obtained through Illumina MiSeq using PCR-amplified 16S rRNA gene fragments. Indications are given for the composition with a frequency of 1% or more except for *Acinetobacter*.

*Enterococcus* bacteria predominated in the original digestive tracts of the three waxworm batches each (Fig. 1A and Table S1). Using the digestive tract mixture of three waxworm batches as an inoculum, bacteria degrading PE or PP fragments were enriched on C_16_ or pristane, respectively, in MP medium (an inorganic medium for terrestrial bacteria) for 24 days at room temperature (16–24 ℃). In these cultures, *Acinetobacter* bacteria predominated (Fig. 1A). At the species level, *Acinetobacter courvalinii* was detected as the most abundant species in both C_16_- and pristane-enriched cultures, at 36.5 and 34.5% of the total bacteria, respectively (Table S2). *Acinetobacter calcoaceticus* NCCB 22016^T^ is almost identical in the 16S rRNA gene sequence to *Acinetobacter pittii* strains ATCC 19004^T^ (99.9% similarity) and CIP 70.29^T^ (99.7%) but is more different from *A. calcoaceticus* ATCC 23055^T^ (98.0%). This group containing *A*. *calcoaceticus* and *A. pittii* was detected as the second most abundant species-level group in *Acinetobacter* in C_16_- and pristane-enriched cultures, at 9.5 and 5.5%, respectively (Table S2). It has been indicated that *Acinetobacter* bacteria, including *A. courvalinii*, *A. pittii*, and *A. calcoaceticus*, degrade *n*-alkanes^43–45^ and that *A. pittii* and *A. calcoaceticus* also degrade isoprenoid-derived branched alkanes (pristane and phytane)^46,47^. These data indicate that *Acinetobacter* bacteria, particularly *A. courvalinii*, *A. calcoaceticus*, and *A. pittii*, could be potent degraders of PE and PP fragments among the digestive tract bacteria of *Galleria* waxworms and could be major degraders of these fragments in the worms.

Regarding the potential PE- and PP-degrading bacteria so far identified from worms, *Bacillus* and *Pseudomonas* bacteria were not found in the C_16_- and pristane-enriched cultures as well as in the original digestive tracts (Fig. 1 and Tables S1 and S2). *Enterobacter* and *Serratia* bacteria were detected abundantly in the C_16_- and pristane-enriched cultures but also in the original digestive tracts of one waxworm batch (batch 3) (Fig. 1 and Tables S1 and S2).

### Alkane-degrading isolates of *Acinetobacter*

Bacteria were isolated from C_16_- and pristane-enriched cultures on MP (medium) plates covered with C_16_ or pristane, respectively. Colonies of different morphologies were selected, and the selected isolates from the C_16_- or pristane-enriched culture were grown in MP medium with C_16_ or pristane, respectively, at 20 ℃ for 3 weeks to confirm the degrading ability. The C_16_- or pristane-degrading isolates were subjected to repetitive extragenic palindromic sequence PCR (rep-PCR) analysis for strain typing. Two C_16_-degrading isolates were obtained from the C_16_-enriched culture and designated as strains Bh10 and Bh12. Two pristane-degrading isolates were also obtained from the pristane-enriched culture and showed the same rep-PCR patterns as strains Bh10 and Bh12 (Fig. S1). Strain Bh10 grew at 4–42 ℃ (optimally at 37 ℃) but not at 50 ℃ and strain Bh12 grew at 4–50 ℃ (optimally at 37 ℃) on LB plates.

The almost full-length 16S rRNA gene sequence (Table 1) indicate that strains Bh10 and Bh12 belong to *A. courvalinii* and to *A. pittii* or *A*. *calcoaceticus*, respectively. *A. pittii* bacteria have been grown at 44 ℃, while *A*. *calcoaceticus* bacteria have not^48^. As strain Bh12 grew up to 50 ℃, this strain was considered to belong to *A. pittii*. Strain Bh10 or its relatives were suggested to be abundant, at 38.1 and 37.7% of total bacterial sequences, and strain Bh12 or its relatives were present in significant amounts, at 0.3 and 0.4%, in C_16_- and pristane-enriched cultures, respectively (Table 1). In the original digestive tracts, strain Bh10 or its relatives were detected (0.3% in one batch), and strain Bh12 or its relatives were not (Table 1). These findings and species-level analyses (Tables S1 and S2) indicate that strains Bh10 and Bh12 can be considered representatives of the main enriched *Acinetobacter* species. Strains Bh10 and Bh12 appeared phylogenetically distant from each other within the genus *Acinetobacter* based on their 16S rRNA gene sequence similarity (96.6%).

**Table 1:** C_16_- and pristane-degrading bacteria enriched on C_16_ or pristane in an inorganic MP medium from the waxworm digestive tracts, analyzed using the 16S rRNA gene sequence.

Isolate	Taxonomy					Abundance*			
(Accession No.)	Closest type strain	Similarity (%)	Worm 1	Worm 2	Worm 3	C_16_	Pristane	Ghostshark Intes	Ghostshark Sur
Bh10	*Acinetobacter courvalinii* ANC3623^T^	99.8%	0%	0%	0.3%	38.1%	37.7%	18.6%	22.1%
(LC739303)			(0/27570)	(0/27960)	(97/29182)	(10610/27847)	(14006/37200)	(4616/24868)	(6950/31405)
Bh12†	*Acinetobacter pittii* ATCC 19004^T^	100%	0%	0%	0%	0.3%	0.4%	0.5%	1.6%
(LC739304)			(0/27570)	(0/27960)	(0/29182)	(77/27847)	(158/37200)	(130/24868)	(498/31405)

* Abundance and numbers (in parentheses) of sequences out of total bacterial sequences from the original waxworm digestive tracts (indicated as Worm 1–3 for waxworm batches 1–3), C_16_- or pristane-enriched culture, and ghostshark intestine (Intes) and body surface (Sur) with 99–100% similarity.

† Also 100% identical to that of *Acinetobacter calcoaceticus* NCCB 22016^T^.

An *Enterobacter* strain showing 99.3% 16S rRNA gene sequence identity to *Enterobacter quasihormaechei* WCHEs120003^T^ (Tables S1 and S2) was isolated from the pristane-enriched culture. A *Serratia* strain was not obtained from the enriched cultures and *Serratia grimesii* NBRC 13537^T^ (Tables S1 and S2) was obtained from NBRC (NITE Biological Resource Center, Japan). The *Enterobacter* isolate and the *Serratia* strain did not degrade C_16_ and pristane in MP medium at 20 ℃ for 3 weeks (Fig. S2).

### *n*-Alkane- and isoprenoid-derived branched-alkane-degrading activities by *Acinetobacter* isolates

**Fig. 2:**
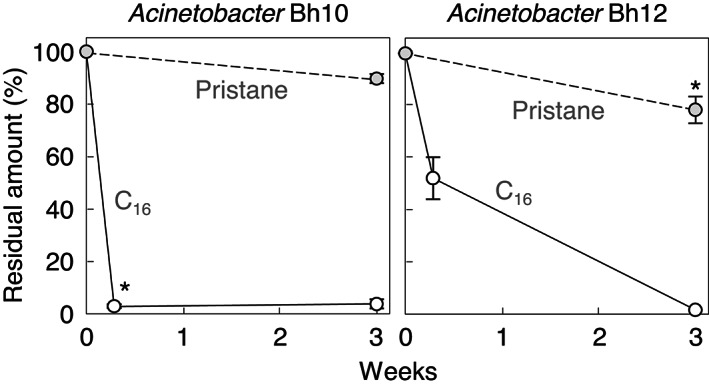
Degradation of C_16_ or pristane (0.04%, vol/vol) in MP medium by isolate Bh10 or Bh12 at 20 ℃. Data were obtained through GC-MS. Non-inoculated sterile samples were similarly incubated and served as controls (100%). These were normalized to equivalent *n*-tetracosane added just prior to extraction. Each value is the mean ± standard error from two or more samples. Residual amounts (%) of C_16_ on day 2 with strain Bh10 were significantly lower (*) than those with strain Bh12 (*p*-value of 0.001 by Student’s *t*-test), while the amounts of pristane on day 21 with strain Bh12 were significantly lower (*) than those with strain Bh10 (*p*-value of 0.08).

The C_16_- and pristane-degrading activities of strains Bh10 and Bh12 in MP medium at 20 ℃ are shown in Fig. 2. Strain Bh10 degraded C_16_ more rapidly than strain Bh12, whereas strain Bh12 degraded pristane more rapidly than strain Bh10. However, strain Bh10 or its relatives were predominantly detected in the pristane-enriched culture, and strain Bh12 or its relatives were not (Table 1 and S2). These results may suggest that strain Bh12 preferentially degraded other carbon sources, from the waxworm digestive tracts, over branched alkanes.

**Fig. 3:**
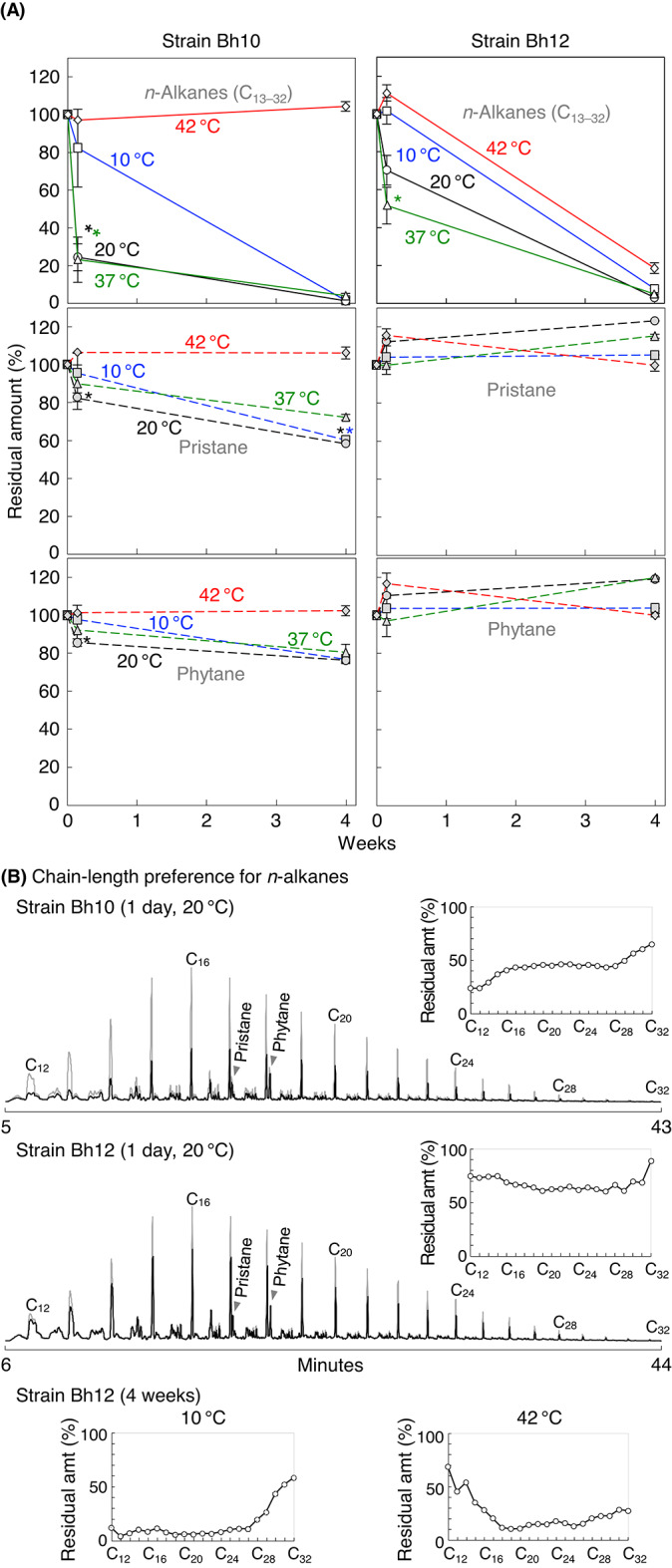
Degradation of *n*-alkanes and branched alkanes (pristane and phytane) in crude oil (0.1%, vol/vol) in MP medium by isolate Bh10 or Bh12 at 10, 20, 37, and 42 ℃. Data were obtained through GC-MS. Non-inoculated sterile samples were similarly incubated and served as controls (100%). These were normalized to 17*α*(*H*),21*β*(*H*)-hopane contained in crude oil. Error bars represent standard errors. With strain Bh10 on day 1, residual amounts (%) of *n*-alkanes at 20–37 ℃ were significantly lower (*) than those at 10 ℃ (*p*-values of 0.05–0.06 by Student’s *t*-test), while the amounts of branched alkanes at 20 ℃ were lower than those at 10 ℃ (*p*-values of 0.14–0.21) and 37 ℃ (*p*-values of 0.28–0.30). On day 21, the amounts of pristane at 10–20 ℃ were significantly lower than those of phytane (*p*-value of 0.01). With strain Bh12 on day 1, residual amounts (%) of *n*-alkanes at 37 ℃ were lower than those at 42, 10, and 20 ℃ (*p*-values of 0.008, 0.05, and 0.16, respectively). Chain-length preference for *n*-alkanes, observed during the degradation (**A**), is shown by extracted ion monitoring chromatograms of m/z 57 (**B**). Data from control samples are shown superimposed in gray in these chromatograms. Residual amounts (amt) of *n*-alkanes are also shown by the inset figures. The amounts at different temperatures by strain Bh12 are also shown. These chain-length preference tendencies for *n*-alkanes (**B**) were observed at least twice.

Degradation of *n*-alkanes and isoprenoid-derived branched alkanes (pristane and phytane) was analyzed using crude oil as the alkane source in MP medium at 10, 20, 37, and 42 ℃ (Fig. 3). Strain Bh10 degraded *n*-alkanes and branched alkanes except at 42 ℃ and most rapidly at 20–37 ℃ and 20 ℃, respectively (Fig. 3A). This shows that branched alkanes were not efficiently degraded at a higher temperature, 37 ℃, at which *n*-alkanes were efficiently degraded. Short *n*-alkanes were preferred at 10–20 ℃ (Fig. 3B and S3), whereas chain-length preference was not observed at 37 ℃ (Fig. S3). Pristane was more degradable than phytane, implying that the isopropyl terminus was more susceptible to oxidation than the ethyl terminus by strain Bh10 (Fig. 3A). Strain Bh12 degraded *n*-alkanes at all the temperatures tested including 42 ℃ and most rapidly at 37 ℃ (Fig. 3A). Branched alkanes were not degraded, potentially due to the presence of other carbon sources in the crude oil (Fig. 3A). Short *n*-alkanes were preferred at 10–20 ℃, whereas non-short *n*-alkanes were preferred at 42 ℃ (Fig. 3B, Fig. S3 for the data at 20 ℃). These chain-length preferences appeared mixed at 20–37 ℃ (Fig. 3B, Fig. S3 for the data at 37 ℃). Thus, at 20 ℃, preference for short *n*-alkanes (Fig. S3) or mixed preference (Fig. 3B) was observed. These data may suggest at least two types of *n*-alkane-degrading enzymes in strain Bh12. Owing to these different degradation characteristics, strains Bh10 and Bh12 could efficiently degrade PE fragments in waxworms.

As has been reported for *Acinetobacter* including *A. pittii* and *A. calcoaceticus*^44,46,47,49^, strains Bh10 and Bh12 were indicated to degrade the aromatic compounds contained in crude oil (Fig. S4). This suggests that the strains may also degrade aromatic structure of aromatic-containing plastics, such as polyethylene terephthalate^50^. At the same time, strains Bh10 and Bh12 were also indicated to degrade 17*α*(*H*),21*β*(*H*)-hopane in the crude oil (Fig. S4), even though it is generally considered highly resistant to biodegradation^51^.

### Liquid PP-degrading activities by *Acinetobacter* isolates

Without the addition of a carbon source, strains Bh10 and Bh12 grew negligibly and substantially, respectively, on MP plates. This could be caused by a carbon and energy source accumulated during preculture. C_16_-, pristane-, and liquid PP-degrading activities on MP plates (growth on these alkanes) were thus analyzed only with strain Bh10 at 10, 20, 37, and 42 ℃. Strain Bh10 grew fastest at 20–37 ℃ on C_16_, 20 ℃ on pristane, and 10–20 ℃ on liquid PP, and did not grow on them at 42 ℃ (Fig. S5), consistent with the results in liquid cultures (Fig. 3A). The data suggest that liquid PP was used as a carbon source and that PE and PP fragments may be preferentially degraded at warm (20–37 ℃) and cold (10–20 ℃) temperatures, respectively, by strain Bh10 (probably *A. courvalinii*). Similarly, liquid PP is preferentially degraded at low temperature by *Alcanivorax*^14^.

**Fig. 4:**
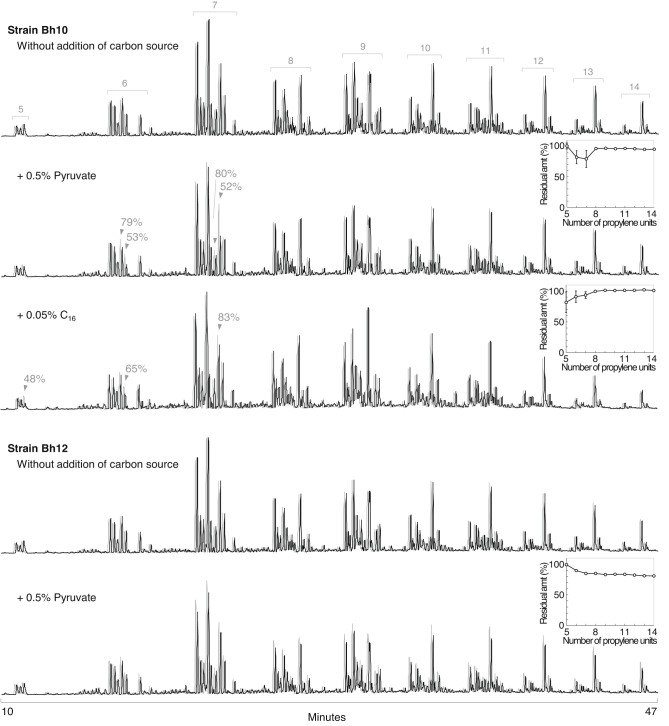
Degradation of liquid PP (0.04%, vol/vol) in MP medium by isolate Bh10 or Bh12 with or without C_16_ or pyruvate at 20 ℃ for 4 weeks. GC-MS total ion chromatograms are shown. Concentrations of C_16_ (vol/vol) or pyruvate (wt/vol) are indicated. PP pentamers to tetradecamers are indicated as numbers above the peaks. Liquid PP in non-inoculated control samples is shown superimposed in gray and at a slightly left position. These are normalized to equivalent *n*-dodecane added just prior to extraction. For strain Bh10, degraded peaks and the residual amounts are indicated. When degradation was observed, the residual amount (amt) of each PP oligomer, using the main peak(s), is also shown as an inset figure. Error bars, representing standard errors from these main peaks of each oligomer, were smaller than the size of the symbols for strain Bh12 with 0.5% pyruvate. Data of strains Bh10 and Bh12 are from Fig. S6 and S7, respectively, and these degradations were confirmed by repeated experiments (Fig. S6 and S7).

Degradation of liquid PP was also examined in MP medium supplemented with or without C_16_ or pyruvate at 20 ℃ for 4 weeks (Fig. 4, S6, and S7). Pyruvate was used because it is a central metabolite probably used by most bacteria and would not be a specific inducer of alkane hydroxylase. By contrast, C_16_ could be an inducer of alkane hydroxylase^22^. Strains Bh10 and Bh12 degraded liquid PP only with the addition of another carbon source, including pyruvate (Fig. 4, S6, and S7), showing that PP was not used as the sole carbon and energy source and that compounds used as the sole carbon and energy source would be required to show the PP-degrading activity. This is consistent with the observation with *Alcanivorax*^14^. Strain Bh10 degraded short PP oligomers, a pentamer to heptamers (Fig. 4 and S6). A pentamer to a heptamer were degraded with 0.05% (vol/vol) C_16_ but not with 0.05% (wt/vol) pyruvate. With 0.5–1% (wt/vol) pyruvate, hexamers and heptamers were degraded. These results may suggest that C_16_ could have induced the expression of another PP-degrading enzyme that degrades the pentamer. Because C_16_ is naturally derived from liquid PP^14^, strain Bh10 could degrade the pentamer without addition of C_16_ in the long term. With 0.2% (vol/vol) C_16_, peaks of PP hexamers became broad and diffuse in the GC-MS chromatogram. Therefore, degradation with a larger quantity of C_16_ was not investigated. Strain Bh12 degraded all the PP oligomers detected, except pentamers, with 0.5–1% (wt/vol) pyruvate (Fig. 4 and S7). Degradation was not observed with 0.05% (vol/vol) C_16_ and 0.05% (wt/vol) pyruvate (Fig. S7). Longer oligomers were preferred (Fig. 4 and S7). The differences in the length preferences of strains Bh10 and 12 indicate that these strains could also efficiently degrade PP fragments in waxworms. Correlation between the chain-length preferences for *n*-alkanes (Fig. 3) and PP oligomers (Fig. 4, S6, and S7) by strains Bh10 and Bh12 at 20 ℃ may support the hypothesis that *n*-alkane hydroxylase could be involved in hydroxylation of the isopropyl terminus of branched alkanes. This is the first report of clear PP biodegradation by terrestrial organisms.

### Degradation of PE and PP films by *Acinetobacter* isolates

**Fig. 5:**
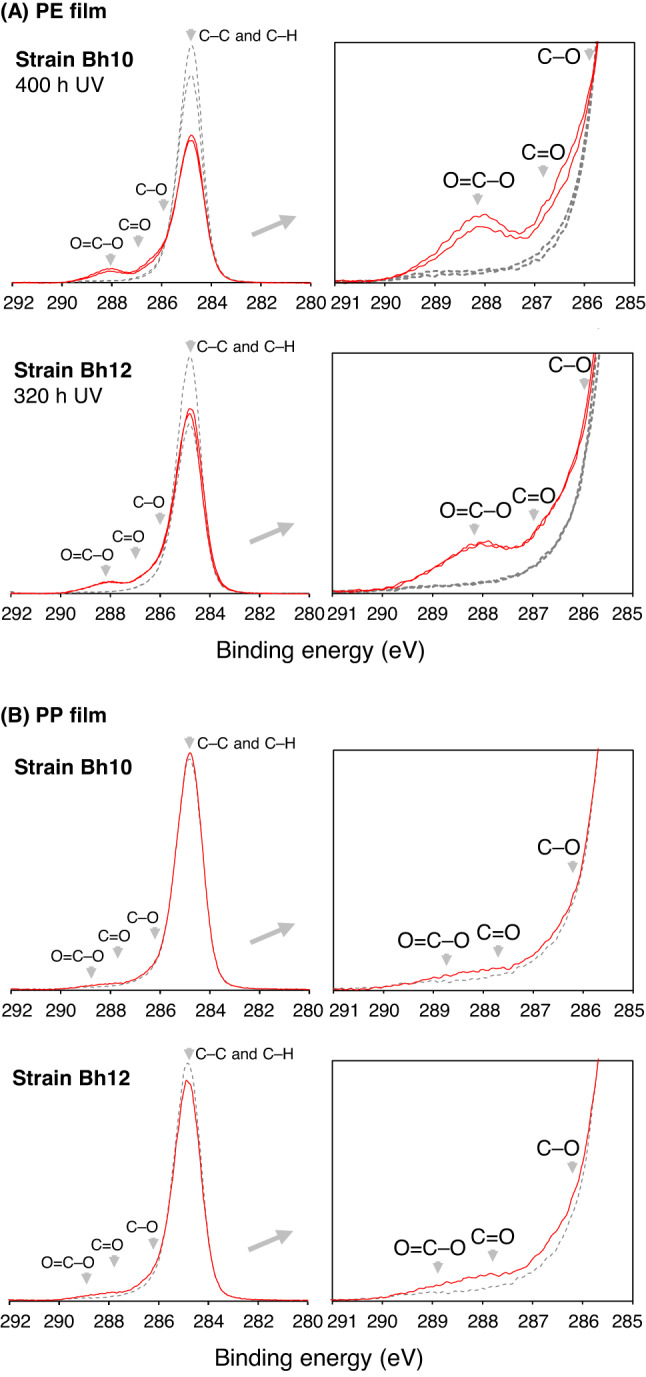
XPS spectra of C1s for PE (**A**) or PP (**B**) film incubated in MP medium with strain Bh10 or Bh12 for 4 weeks. PE film was incubated at 37 ℃ with 0.021% (vol/vol) C_16_, while PP film was incubated at 20 ℃ with 0.5% (wt/vol) pyruvate. The spectra for films incubated in the same way without strains (controls) are shown as dashed lines. The spectra for PE film irradiated with UV for 320 or 400 h and PP film irradiated with UV for 75 h are shown. Two points were measured on each PE film, while areas (500 μm × 300 μm) were measured on each PP film. Figures on the right are expansions of the figures on the left. Data are from Fig. S8 and S10. Arrow heads indicate the peaks of resolved spectra (Fig. S9 and S11) for the linkages.

Degradation of UV-irradiated PE films by strain Bh10 or Bh12 was examined in MP medium supplemented with 0.021% (vol/vol) C_16_ at 37 ℃ for 4 weeks. Weight loss of the films was observed with each strain, up to 4% each: for strain Bh10, 0, 4, 4% for 320, 340, and 400 h UV-irradiated films, respectively, while for strain Bh12, 4 and 0% for 320 and 340 h UV-irradiated films, respectively. The surfaces of the PE films which weight was lost at 4% (400 h UV-irradiated film for strain Bh10 and 320 h UV-irradiated film for strain Bh12) were analyzed using X-ray photoelectron spectroscopy (XPS) (Fig. 5A, S8, and S9). The C1s XPS spectra showed a new peak and shoulder at approximately 288 and 287 eV for the PE films, indicating the formation of O = C–O and C = O linkages, respectively. The O = C–O and C = O linkages were implied to be increased with strain Bh10 by 7.5 and 1.6 atomic% and with strain Bh12 by 5.0 and 1.7 atomic%, respectively, of the total C compared with the controls (Fig. S9). The total oxygen-linked carbon (O = C–O, C = O, and C–O) was implied to be increased with strain Bh10 by 9.5 atomic% and with strain Bh12 by 5.7 atomic% of the total C compared with the controls (Fig. S9). The O1s XPS spectra supported film oxidation (Fig. S8). The O/C atomic ratio of the film increased with strain Bh10 and could have increased slightly with strain Bh12 (Table S3).

Degradation of UV-irradiated PP films (solid PP) by strain Bh10 or Bh12 was also examined in MP medium supplemented with 0.5% (wt/vol) pyruvate at 20 ℃ for 4 weeks, and the surfaces of the films were analyzed using XPS (Fig. 5B, S10, and S11). The C1s XPS spectra for 75 h UV-irradiated films showed new shoulders at approximately 289 and 288 eV, indicating the formation of O = C–O and C = O linkages, respectively, with each strain and at approximately 286 eV, indicating the formation of C–O linkage, with strain Bh12 (for 74 h UV-irradiated films, the linkage formation was not clear with each strain). The weight loss was not observed with each strain. The O = C–O, C = O, and C-O linkages were implied to be increased with strain Bh10 by 0.6, 0.8, and 0.1 atomic% and with strain Bh12 by 0.7, 1.6, and 1.3 atomic%, respectively, of the total C compared with the control (Fig. S11). The O1s XPS spectra (Fig. S10) and O/C atomic ratio (Table S4) supported film oxidation. These results indicate that the PE and PP films were enzymatically oxidated by strains Bh10 and Bh12, but that fragmentation and fluidity are required for their efficient biodegradation, as has been shown for PP films with *Alcanivorax*^14^.

### Abundance of the *Acinetobacter* in the intestine and on the body surface of a purple ghostshark from the deep sea

The bacterial compositions in the intestine and on the body surface of a purple ghostshark of *Hydrolagus purpurescens* obtained from the deep sea, during another research, were unexpectedly found to be similar to those in the C_16_- and pristane-enriched cultures (Fig. 1A and B; Table S2 and S5). *A. courvalinii* bacteria and the *A. calcoaceticus* and *A. pittii* group bacteria were found to be abundant, at 19% and 3% of the total bacteria in the intestine, and 22% and 6% on the body surface, respectively (Table 1 and S5). In the intestine, *A. courvalinii* was found to be the most abundant species and the *A. calcoaceticus* and *A. pittii* group was found to be the second most abundant species-level group in *Acinetobacter* (Table S5). Ghostsharks characteristically contain much diacyl glyceryl ethers in their liver^41,42^. *Acinetobacter* metabolizes alkyl chains of ethers^52^, which are generally highly resistant to biodegradation^53^. These data suggest that *A. courvalinii*, *A. calcoaceticus*, and *A. pittii* bacteria may have been enriched on the alkyl chains in the ghostshark intestine. The abundance of these bacteria on the body surface (Table 1 and S5) may also suggest the presence of much alkyl chains on the body surface. The reason of the similar bacterial composition to that from a terrestrial environment could be due to the homeostasis by the marine organism. PE and PP could become fragmented and fluid with much alkyl chains on the ghostsharks, and the fragmented fluid PE and PP could be biodegraded by the abundant *Acinetobacter* bacteria. Overall, the results suggest that *Acinetobacter* bacteria, especially *A. courvalinii*, *A. calcoaceticus*, and *A. pittii*, could be potent degraders of alkyl chains, including PE and PP fragments, among the digestive tract bacteria of some terrestrial and marine organisms, including PE- and PP-ingesting worms.

## Materials and methods

### Waxworms

Living *Galleria mellonella* waxworms grown in terrestrial environments and supplied as fishing bait were obtained. Three batches of the waxworms were obtained: two batches from BlueEarthFishing Co., Ltd. (Shizuoka, Japan) in Sep. and Oct. 2018 and another from Charm Co., Ltd. (Gunma, Japan) in Nov. 2018. Waxworms were stored at 4 ℃ before use.

### *n*-Hexadecane- and pristane-enriched cultures

Digestive tracts from three living waxworms in the three batches each were ground using an ice-cold mortar and pestle. A portion of the homogenate from the three batches each was dried at 50 ℃ for 6 h for preventing DNA degradation and stored at – 80 ℃ for community analysis.

The remaining homogenates from the three batches each were mixed. One half of this homogenate mixture was inoculated into 10 ml MP medium supplemented with 0.25 µl C_16_ (Nacalai, Japan). MP medium was the same composition as that reported previously^54^ except that 0.015 g/L FeCl_2_·4H_2_O was used instead of 0.02 g/L FeCl_3_·6H_2_O. The other half of the homogenate mixture was inoculated into 10 ml MP medium with 0.25 µl pristane (Synthetic; Wako, Japan). The C_16_- and pristane-enriched cultures were incubated with shaking at room temperature (16–24 ℃) at which waxworms grow. When these oils (C_16_ and pristane) disappeared (on the 20th day after starting the cultivation), 0.25 µl C_16_ or pristane was added to C_16_- or pristane-enriched culture, respectively. When these oils again disappeared (on the 21st day for C_16_-enriched culture and on the 23rd day for pristane-enriched culture), C_16_- or pristane-enriched culture was diluted and plated on MP (medium) plate (1.5%, wt/vol, agar; purified for microbial cultures; Nacalai; 9 cm in diameter) covered with 2 µl C_16_ or pristane to isolate C_16_- or pristane-degrading bacteria, respectively. The plates were incubated at 20 ℃ for 6 days for C_16_-enriched culture and for 7–8 days for pristane-enriched culture. Isolates Bh10 and Bh12 were deposited in NBRC under numbers NBRC 115945 and NBRC 115946, respectively.

On the 22nd day, 0.25 µl C_16_ was again added to the C_16_-enriched culture. For bacterial community analysis, 4.8 ml C_16_-enriched culture and 3.6 ml pristane-enriched culture were separately dried by vacuum evaporation at room temperature (16–24 ℃) and stored at – 80 ℃ on the 24th day.

### Bacterial community composition

The procedure from DNA extraction from the dried samples (original digestive tracts from three waxworm batches and C_16_- and pristane-enriched cultures) to acquisition of bacterial 16S rRNA sequences was carried out as described previously^14^. The sequencing was conducted on MiSeq (Illumina, CA, USA) using the 16S rRNA gene fragments amplified with primers with the priming regions of Pro341F and Pro805R^55^. Sequences that had quality value scores of 20 or more for more than 99% of the sequence were selected^14^. The non-chimeric sequences obtained just before removing the non-bacterial sequences were deposited in GenBank/EMBL/DDBJ: 27,571, 27,960, and 29,182 sequences from the digestive tracts of three waxworm batches each under accession numbers TAAI01000001-TAAI01027571, TAAJ01000001-TAAJ01027960, and TAAK01000001-TAAK01029182, respectively, and 27,848 and 37,202 sequences from C_16_- and pristane-enriched cultures under accession numbers TAAL01000001-TAAL01027848 and TAAM01000001-TAAM01037202, respectively.

Bacterial 16S rRNA sequences of 27,570, 27,960 and 29,182 sequences from the three waxworm batches each and 27,847 and 37,200 sequences from C_16_- and pristane-enriched cultures, respectively, were analyzed for community analysis. The sequence was assigned to the genus using the RDP Classifier^56^ with a confidence threshold of 80% and to species using BLAST^57^.

A purple ghostshark of *Hydrolagus purpurescens* was obtained from Suruga Bay, Shizuoka, Japan (34°51′28″ N, 138°36′20″ E, depth 1300–1600 m) on October 22, 2021. The body surface was rubbed with a 0.22-µm pore size membrane (Express PLUS, Millipore) to collect the bacteria on the surface. The intestine was also obtained. The body surface sample (a membrane) and intestinal sample (a portion of the intestine) were immediately stored at – 80 ℃. Every step from fish acquisition to sample storage was performed on the same day. Bacterial 16S rRNA sequences were obtained from samples in the same way as described above. The non-chimeric 31,434 and 25,063 sequences from the body surface and intestinal samples, obtained just before removing the non-bacterial sequences, were deposited in GenBank/EMBL/DDBJ under accession numbers TAAV01000001-TAAV01031434 and TAAW01000001-TAAW01025063, respectively. Bacterial 16S rRNA sequences, 31,405 and 24,868 sequences from the body surface and intestinal samples, respectively, were analyzed in the same manner as described above for community analysis.

### Degradation of alkanes and crude oil and GC-MS analysis

C_16_-, pristane-, liquid PP- or crude-oil-degrading activity was examined in 2 ml MP medium supplemented with 0.8 µl C_16_, 0.8 µl pristane, 0.8 µl liquid PP, or 2 µl crude oil (heat-treated Arabian light crude oil; a gift from ENEOS Corporation, Japan)^19^. C_16_- and/or pristane-degrading activities were investigated with all isolates and *S. grimesii* NBRC 13537^T^ (obtained from NBRC), while liquid PP- and crude-oil-degrading activities were investigated with isolates Bh10 and Bh12. The liquid PP was prepared by the San-ei Kogyo Corporation by heating solid PP (containing additives at less than 3%, wt/wt) at 370 ℃^58^. The resultant liquid PP consisted of terminal monoolefins, PP, and terminal diolefins as the dominant, second dominant, and minor components, respectively, as confirmed by the San-ei Kogyo Corporation using GC-MS and partly NMR. For liquid PP-degrading activity, the indicated amounts of sodium pyruvate (wt/vol) or C_16_ (vol/vol) were added. Each bacterium freshly grown on an LB plate (Miller, Nacalai) was inoculated into the medium using a toothpick. For pristane-degrading activity with isolates and *S. grimesii* NBRC 13537^T^, the bacterium freshly grown on an MP plate covered with 2 µl pristane was also inoculated. C_16_- and pristane-containing cultures were incubated for 3 weeks and liquid PP-containing cultures were incubated for 4 weeks with shaking at 20 ℃. Crude-oil-containing cultures were incubated for 4 weeks with shaking at 10, 20, 37, or 42 ℃. Non-inoculated sterile samples were similarly incubated and served as controls. Pyruvate or C_16_ was not added to the control samples for liquid PP-degrading activity.

Hydrocarbons were extracted twice from the cultures by vigorous shaking with 2 ml *n*-heptane for 1 min. The 0.01 mg *n*-tetracosane for C_16_- or pristane-containing cultures and 0.027 µl *n*-dodecane for liquid PP-containing cultures were added immediately before extraction as an internal standard to evaluate degradation. Sodium sulfate was added to the *n*-heptane extracts to dehydrate them, and the supernatants were concentrated to approximately 150 µl for C_16_-, pristane- and liquid PP-supplemented cultures or 67 µl for crude-oil-supplemented cultures by N_2_ purging. The concentrated extracts were analyzed by GC–MS using a 6890N gas chromatograph as previously described^19^, unless stated otherwise. To investigate the degradation of C_16_, pristane, and liquid PP, their peak areas obtained with GC-MS total ion monitoring were normalized by the peak area for *n*-tetracosane or *n*-dodecane and compared to those from the controls. To investigate crude oil degradation, the peak areas obtained with GC-MS extracted ion monitoring were normalized by the peak area for 17*α*(*H*),21*β*(*H*)-hopane^51^. The degradation of each liquid PP oligomer (pentamer to tetradecamer) was compared using the main peak(s).

### DNA analysis of isolates

Rep-PCR for genomic fingerprints of bacteria, performed using primers REP1R-I and REP2-I^59^, and 16S rRNA gene sequencing of the isolates were conducted as described previously^19^. The 16S rRNA gene fragment sequences were assigned using BLAST^57^.

### Growth temperature and utilization of alkanes as sole carbon sources

Growth temperatures of isolates Bh10 and Bh12 were tested at 4, 10, 15, 20, 28, 37, 42, and 50 ℃ on LB plates for 4 weeks. Growth was assessed visually.

To investigate utilization of alkanes as sole carbon sources, isolates Bh10 and Bh12 pregrown on an LB plate were plated on MP plates covered with 3 µl C_16_, pristane or liquid PP. The plates were incubated at 10, 20, 37 or 42 ℃ for 14 days (for pristane cultures) or 18 days (for C_16_ and liquid PP cultures). Growth on MP plates that were not covered with C_16_, pristane or liquid PP was also tested at 28 ℃ for 16 days as controls. Growth was assessed visually.

### Degradation of PE and PP films and XPS analysis

A low-density PE film (Yutaka Finepack Co., Ltd., Japan) that did not contain any additives and PP film of Torayfan no. 3501 (Toray Advanced Film Co., Ltd, Japan) that contained additives at less than 0.1% (wt/wt) were used. The PE films were UV-irradiated for 320, 340, or 400 h and PP films were UV-irradiated for 74 or 75 h at 254 nm with a UV Crosslinker CL-1000 (Funakoshi, Japan) until they cracked easily when pinched. During UV-irradiation, they floated on Milli-Q water (Merck) 11.5 cm from the inside bottom.

Isolate Bh10 or Bh12 freshly grown on an LB plate was inoculated using a toothpick into 4.8 ml MP medium supplemented with 1 µl C_16_ and 3.9–5.6 mg UV-irradiated PE films or with 0.5% (wt/vol) sodium pyruvate and 2.5–3.4 mg UV-irradiated PP films. The culture was incubated for 4 weeks with shaking at 37 ℃ for PE films and at 20 ℃ for PP films. Non-inoculated sterile samples were similarly incubated and served as controls. The films were then treated to remove biofilms as described previously^14^. The dry weight was measured with an ionizer (STABLO-AP, Shimadzu, Japan). The hydroxyl, carbonyl, and carboxyl groups on the PE and PP film surfaces were quantified using XPS as described previously^14^. For PE films, points were measured, and for PP films, areas (500 μm ⨯ 300 μm) were measured.

## Supplementary Information

Below is the link to the electronic supplementary material.

Supplementary Material 1

## Data Availability

The 27571, 27960, and 29182 sequences from the digestive tracts of three waxworm batches were deposited in GenBank/EMBL/DDBJ under accession numbers [TAAI01000001](https:/www.ncbi.nlm.nih.gov/nuccore/TAAI01000001) - [TAAI01027571](https:/www.ncbi.nlm.nih.gov/nuccore/TAAI01027571) , [TAAJ01000001](https:/www.ncbi.nlm.nih.gov/nuccore/TAAJ01000001) - [TAAJ01027960](https:/www.ncbi.nlm.nih.gov/nuccore/TAAJ01027960) , and [TAAK01000001](https:/www.ncbi.nlm.nih.gov/nuccore/TAAK01000001) - [TAAK01029182](https:/www.ncbi.nlm.nih.gov/nuccore/TAAK01029182) , respectively, 27848 and 37202 sequences from C_16_- and pristane-enriched cultures under accession numbers [TAAL01000001](https:/www.ncbi.nlm.nih.gov/nuccore/TAAL01000001) - [TAAL01027848](https:/www.ncbi.nlm.nih.gov/nuccore/TAAL01027848) and [TAAM01000001](https:/www.ncbi.nlm.nih.gov/nuccore/TAAM01000001) - [TAAM01037202](https:/www.ncbi.nlm.nih.gov/nuccore/TAAM01037202) , respectively, and 31434 and 25063 sequences from the body surface and intestinal samples under accession numbers [TAAV01000001](https:/www.ncbi.nlm.nih.gov/nuccore/TAAV01000001) - [TAAV01031434](https:/www.ncbi.nlm.nih.gov/nuccore/TAAV01031434) and [TAAW01000001](https:/www.ncbi.nlm.nih.gov/nuccore/TAAW01000001) - [TAAW01025063](https:/www.ncbi.nlm.nih.gov/nuccore/TAAW01025063) , respectively.
