# Supplementary material for: Acinetobacter bacteria could be potent degraders of fragmented polyethylene and polypropylene among the digestive tract bacteria of Galleria waxworms
Source: Sci Rep. 2026 Mar 9;16:12794. doi: 10.1038/s41598-026-40931-7 (PMC13096178; doi:10.1038/s41598-026-40931-7)
Supplement: Supplementary file 1 — Supplementary Material 1 [file 41598_2026_40931_MOESM1_ESM.pdf]

Table S1. Bacterial community composition at the species level in the digestive tract of *Galleria* waxworms
Twenty randomly selected 16S rRNA gene fragment sequences from each of the three waxworm batches were analyzed by BLAST.

No. of sequences	Closest species (compared to the type strain)	Similarity
Waxworm batch 1		
10 (50%)	*Enterococcus mundtii*	99.3–100%
1 (5%)	*Enterococcus durans*, *Enterococcus faecium*, *Enterococcus hirae*, *Enterococcus mundtii*, *Enterococcus ratti*, *Enterococcus villorum*	99.5%
7 (35%)	*Enterococcus casseliflavus*, *Enterococcus innesii*	98.9–100%
2 (10%)	*Staphylococcus xylosus*	99.1–99.8%
Waxworm batch 2		
13 (65%)	*Enterococcus mundtii*	99.1–100%
2 (10%)	*Enterococcus faecalis*	99.8%
1 (5%)	*Enterococcus casseliflavus*, *Enterococcus innesii*	100%
4 (20%)	*Corynebacterium glyciniphilum*	99.8–100%
Waxworm batch 3		
3 (15%)	*Enterococcus casseliflavus*, *Enterococcus innesii*	98.9–100%
2 (10%)	*Enterococcus canintestini*, *Enterococcus dispar*, *Enterococcus saigonensis*	99.8–100%
1 (5%)	*Enterococcus mundtii*	99.8%
3 (15%)	*Enterobacter asburiae*, *Enterobacter cloacae*, *Enterobacter hormaechei*, *Enterobacter quasihormaechei*	99.8–100%
1 (5%)	*Enterobacter mori*, *Enterobacter quasiroggenkampii*, *Enterobacter sichuanensis*	99.5%
1 (5%)	*Enterobacter mori*, *Enterobacter quasiroggenkampii*, *Enterobacter sichuanensis*	97.7%
3 (15%)	*Curtobacterium plantarum*	99.1–99.3%
3 (15%)	*Pantoea vagans*	99.8–100%
1 (5%)	*Rahnella bonaserana*, *Rahnella woolbedingensis* *Serratia fonticola*	99.3%
1 (5%)	*Serratia grimesii, Serratia liquefaciens*	100%
1 (5%)	*Vagococcus fluvialis*	99.8%

Table S2. Bacterial community composition at the species level in C_{16}- and pristane-enriched cultures of the waxworm digestive tract bacteria

Two hundred randomly selected 16S rRNA gene fragment sequences were analyzed by BLAST. ATCC, strain ATCC 23055; NCCB, strain NCCB 22016.

No. of sequences	Closest species (compared to the type strain)	Similarity
C_{16}-enriched		
73 (**36.5%**)	***Acinetobacter courvalinii***	98.8–99.3%
3 (1.5%)	*Acinetobacter courvalinii*	97.0–98.4%
1 (0.5%)	*Acinetobacter courvalinii*	95.8%
1 (0.5%)	*Acinetobacter courvalinii*, *"Alkanindiges hydrocarboniclasticus"*	94.4%
19 (**9.5%**)	***Acinetobacter calcoaceticus*** (ATCC)	98.8–99.3%
1 (0.5%)	*Acinetobacter calcoaceticus* (ATCC)	98.4%
49 (24.5%)	*Chryseobacterium ginsenosidimutans*, *Chryseobacterium zeae*	98.6–99.5%
3 (1.5%)	*Chryseobacterium ginsenosidimutans*, *Chryseobacterium zeae*	97.9–98.3%
8 (4.0%)	*Serratia grimesii*, *Serratia liquefaciens*, *Serratia proteamaculans*	98.6–99.8%
3 (1.5%)	*Serratia fonticola*	99.3–99.8%
10 (5.0%)	*Rahnella bonaserana*, *Rahnella woolbedingensis*<br>*Serratia fonticola*	98.8–99.5%
7 (3.5%)	*Enterobacter asburiae*, *Enterobacter cloacae*, *Enterobacter hormaechei*, *Enterobacter quasihormaechei*	98.8–99.8%
1 (0.5%)	*Enterobacter asburiae*, *Enterobacter cloacae*, *Enterobacter hormaechei*, *Enterobacter quasihormaechei*<br>*Klebsiella pneumoniae*, *Klebsiella quasipneumoniae*	98.1%
2 (1.0%)	*Enterobacter mori*, *Enterobacter quasiroggenkampii*, *Enterobacter sichuanensis*	99.8%
7 (3.5%)	*Mitsuaria noduli*	98.8–99.8%
4 (2.0%)	*Brevibacterium celere*, *Brevibacterium epidermidis*, *Brevibacterium iodinum*, *Brevibacterium limosum*, *Brevibacterium pigmentatum*, *Brevibacterium sanguinis*, *Brevibacterium sediminis*	99.3–99.8%
1 (0.5%)	*Brevibacterium aurantiacum*	99.0%
1 (0.5%)	*Enterococcus canintestini*, *Enterococcus dispar*, *Enterococcus saigonensis*	97.4%
1 (0.5%)	*Enterococcus faecalis*	99.8%
1 (0.5%)	*Enterococcus mundtii*	99.8%
3 (1.5%)	*Vagococcus fluvialis*	99.5–99.8%
1 (0.5%)	*Corynebacterium glyciniphilum*	99.8%
Pristane-enriched		
69 (**34.5%**)	***Acinetobacter courvalinii***	98.6–99.3%
5 (2.5%)	*Acinetobacter courvalinii*	97.0–98.4%
10 (**5.0%**)	***Acinetobacter calcoaceticus*** (ATCC)	98.8–99.5%
1 (**0.5%**)	***Acinetobacter calcoaceticus*** (NCCB), ***Acinetobacter pittii***	99.8%
44 (22.0%)	*Chryseobacterium ginsenosidimutans*, *Chryseobacterium zeae*	98.6–99.3%
2 (1.0%)	*Chryseobacterium ginsenosidimutans*, *Chryseobacterium zeae*	97.4–98.3%
29 (14.5%)	*Serratia grimesii*, *Serratia liquefaciens*, *Serratia proteamaculans*	98.6–99.8%
6 (3.0%)	*Serratia fonticola*	99.1–99.8%
15 (7.5%)	*Rahnella bonaserana*, *Rahnella woolbedingensis*<br>*Serratia fonticola*	98.9–99.5%
7 (3.5%)	*Enterobacter asburiae*, *Enterobacter cloacae*, *Enterobacter hormaechei*, *Enterobacter quasihormaechei*	99.3–99.8%

1 (0.5%)	*Enterobacter asburiae*, *Enterobacter cloacae*, *Enterobacter hormaechei*, *Enterobacter quasihormaechei* *Klebsiella pneumoniae*, *Klebsiella quasipneumoniae*	99.1%
3 (1.5%)	*Vagococcus fluvialis*	99.5%
1 (0.5%)	*Brevibacterium celere*, *Brevibacterium epidermidis*, *Brevibacterium iodinum*, *Brevibacterium limosum*, *Brevibacterium pigmentatum*, *Brevibacterium sanguinis*, *Brevibacterium sediminis*	99.5%
1 (0.5%)	*Brevibacterium antiquum*, *Brevibacterium aurantiacum*, *Brevibacterium renqingii*	98.8%
2 (1.0%)	*Rhodopseudomonas boonkerdii*, *Rhodopseudomonas pentothenatexigens*, *Rhodopseudomonas thermotolerans*	97.8–98.0%
1 (0.5%)	*Corynebacterium glyciniphilum*	99.8%
1 (0.5%)	*Sphingomonas adhaesiva*, *Sphingomonas insulae*, *Sphingomonas kyungheensis*, *Sphingomonas palmae*, "*Sphingomonas parva*", *Sphingomonas populi*	98.8%
1 (0.5%)	*Nitriliruptor alkaliphilus*	94.3%
1 (0.5%)	*Gordonia humi*	98.8%

Table S3. C, N, and O atomic composition analyzed by XPS of PE films in Fig. S4
The O/C atomic ratio is expressed as the mean ± standard error of the two points.

PE film		Atomic%			O/C atomic ratio
		C	N	O	
Without strain Bh10, 400 h UV (control)					
	Point 1	94.4	–	5.6	0.05 ± 0.01
	Point 2	96.5	–	3.5	
With strain Bh10, 400 h UV					
	Point 1	83.2	6.0	10.9	0.12 ± 0.01
	Point 2	84.0	6.0	10.0	
Without strain Bh12, 320 h UV (control)					
	Point 1	88.7	–	11.3	0.11 ± 0.01
	Point 2	90.4	0.5	9.1	
With strain Bh12, 320 h UV					
	Point 1	84.8	4.6	10.6	0.13 ± 0.00
	Point 2	84.9	4.5	10.7	

Table S4. C, N, and O atomic composition analyzed by XPS of PP films in Fig. S6
Areas (500 μm × 300 μm) were measured.

PP film	Atomic%			O/C atomic ratio
	C	N	O	
Without strains (control)	94.7	–	5.3	0.056
With strain Bh10	91.9	1.0	7.1	0.077
With strain Bh12	91.0	1.3	7.6	0.084

Table S5. Bacterial community composition at the species level in the intestine and on the body surface of a purple ghostshark Two hundred randomly selected 16S rRNA gene fragment sequences were analyzed by BLAST. Species names are not described for the genera with a frequency of 0.5%. ATCC, strain ATCC 23055; NCCB, strain NCCB 22016.

No. of sequences	Closest species (compared to the type strain)	Similarity
Intestine		
23 (**11.5%**)	***Acinetobacter courvalinii***	98.6–99.3%
3 (1.5%)	*Acinetobacter courvalinii*	98.4%
6 (**3.0%**)	***Acinetobacter calcoaceticus*** (ATCC)	99.1–99.3%
13 (6.5%)	*Serratia grimesii*, *Serratia liquefaciens*, *Serratia proteamaculans*	98.9–99.8%
1 (0.5%)	*Serratia grimesii*	95.1%
2 (1.0%)	*Serratia fonticola*	99.3–99.5%
10 (5.0%)	*Rahnella bonaserana*, *Rahnella woolbedingensis*<br>*Serratia fonticola*	98.8–99.5%
1 (0.5%)	*Rahnella bonaserana*, *Rahnella woolbedingensis*	97.3%
11 (5.5%)	*Chryseobacterium ginsenosidimutans*, *Chryseobacterium zeae*	98.8–99.3%
10 (5.0%)	*Arcobacter antarcticus*, *Arcobacter nitrofigilis*	96.0–97.0%
1 (0.5%)	*Thioprofundum lithotrophicum*	95.3%
8 (4.0%)	*Thiohalomonas denitrificans*, *Thiohalomonas nitratireducens*<br>*Thiohalophilus thiocyanatoxydans*<br>*Thioprofundum hispidum*, *Thioprofundum lithotrophicum*	90.9–93.5%
4 (2.0%)	*Desulfonatronum parangueonense*, *Desulfonatronum thiosulfatophilum*	85.5–86.9%
4 (2.0%)	*Enterobacter asburiae*, *Enterobacter cloacae*, *Enterobacter hormaechei*,<br>*Enterobacter quasihormaechei*<br>*Klebsiella quasipneumoniae*	98.4–99.8%
4 (2.0%)	*Halioglobus maricola*, *Halioglobus pacificus*	92.0–96.3%
3 (1.5%)	*Moritella abyssi*	99.1%
1 (0.5%)	*Moritella viscosa*	98.4%
1 (0.5%)	*Wenzhouxiangella limi*	90.9%
3 (1.5%)	*Wenzhouxiangella marina*	91.4–92.8%
3 (1.5%)	*Mariniblastus fucicola*	92.6–93.1%
3 (1.5%)	"*Pelagibacter communis*"	98.5–99.5%
3 (1.5%)	*Vicingus serpentipes*	87.2–94.1%
1 (0.5%)	*Colwellia psychrerythraea*	99.1%
1 (0.5%)	*Colwellia hornerae*	98.4%
2 (1.0%)	*Ilumatobacter fluminis*	96.5%
1 (0.5%)	*Lacinutrix algicola*	98.8%
1 (0.5%)	*Lacinutrix undariae*	96.7%
2 (1.0%)	*Parahaliea aestuarii*	95.1–95.6%
1 (0.5%)	*Polaribacter pectinis*, *Polaribacter haliotis*	98.3%
1 (0.5%)	*Polaribacter porphyrae*	95.0%
2 (1.0%)	*Pontimicrobium aquaticum*	96.9%
2 (1.0%)	*Pseudemcibacter aquimaris*	97.8–98.5%
2 (1.0%)	*Psychrobium conchae*	97.9–98.4%
2 (1.0%)	*Roseibacillus ponti*	87.4–88.6%
1 (0.5%)	*Geobacter metallireducens*	86.6%
1 (0.5%)	*Geobacter sulfurreducens*	80.8%
4 (0.5% each)	*Clostridium*, *Lutimonas*, *Paenihalocynthiibacter* and *Zongyanglinia* spp.	98.8–99.3%
9 (0.5% each)	*Croceibacterium*, *Neptuniibacter*, "*Parasynechococcus*", *Psychroserpens*, *Sphingomonas*,<br>*Cochleicola*, *Heliomarina*, *Marimicrobium* and *Sedimentitalea* spp.	97.2–98.5%

6 (0.5% each)	*Agarilytica*, *Labilibaculum*, *Roseovarius*, *Tenacibaculum*, *Marimonas* (or *Pseudoponticoccus*) and *Urechidicola* spp.	95.0–96.8%
21 (0.5% each)	*Actinomarinicola*, *Aliikangiella*, *Arenicella*, *Desulfocastanea*, *Desulfofaba*, *Desulfogranum*, *Marinobacter*, *Marnyiella*, "*Nitrospina*", *Oceanospirillum*, *Pseudohongiella*, *Putridiphycobacter*, *Salinirepens*, *Sedimenticola*, *Seongchinamella*, *Solirubrobacter*, *Spongiispira*, *Syntrophus*, *Temperatibacter*, *Thioalkalivibrio* and *Woeseia* spp.	90.0–94.9%
22 (0.5% each)	*Adhaeretor*, *Breznakia*, "*Desulfohalophilus*", *Desulfovibrio*, *Dictyoglomus*, *Flavilitoribacter*, *Fulvivirga*, *Kangiella*, *Luteitalea* (or *Syntrophotalea*), *Nitrosophilus*, *Phaselicystis*, *Pseudobacteriovorax*, *Puteibacter*, *Reichenbachiella*, *Roseimaritima*, *Stanieria*, *Streptomyces*, *Tepidiforma*, *Thalassolituus*, *Thalassotalea*, *Thiovibrio* and *Vicinamibacter* spp.	83.5–89.9%
Body surface		
86 (43.0%)	*Serratia grimesii*, *Serratia liquefaciens*, *Serratia proteamaculans*	99.1–100%
3 (1.5%)	*Serratia grimesii*, *Serratia liquefaciens*	97.2–98.3%
1 (0.5%)	*Serratia fonticola*, *Serratia grimesii*, *Serratia liquefaciens*	98.1%
1 (0.5%)	*Rahnella bonaserana*, *Rahnella woolbedingensis*<br>*Serratia fonticola*	99.3%
41 (**20.5%**)	***Acinetobacter courvalinii***	98.6–99.3%
3 (1.5%)	*Acinetobacter courvalinii*	96.5–98.4%
1 (**0.5%**)	***Acinetobacter courvalinii***, *Acinetobacter proteolyticus*	98.8%
7 (**3.5%**)	***Acinetobacter calcoaceticus*** (ATCC)	99.1–99.3%
4 (**2.0%**)	***Acinetobacter calcoaceticus*** (NCCB), ***Acinetobacter pittii***	99.5–99.8%
12 (6.0%)	*Enterobacter asburiae*, *Enterobacter cloacae*, *Enterobacter hormaechei*, *Enterobacter quasihormaechei*<br>*Klebsiella quasipneumoniae*	99.1–99.8%
1 (0.5%)	*Enterobacter asburiae*, *Enterobacter quasihormaechei*	95.3%
13 (6.5%)	*Vagococcus fluvialis*	99.3–99.8%
5 (2.5%)	*Brevibacterium antiquum*, *Brevibacterium aurantiacum*	98.5–99.5%
4 (2.0%)	*Rhodopseudomonas boonkerdii*, *Rhodopseudomonas pentothenatexigens*, *Rhodopseudomonas thermotolerans*	97.8–98.0%
1 (0.5%)	*Enterococcus casseliflavus*, *Enterococcus innesii*	99.5%
2 (1.0%)	*Enterococcus xiangfangensis*	99.5–99.8%
3 (1.5%)	*Leucobacter aerolatus*, *Leucobacter chromiireducens*	98.3–99.0%
2 (1.0%)	*Chryseobacterium ginsenosidimutans*, *Chryseobacterium zeae*	99.1%
2 (1.0%)	*Gordonia humi*	98.5–99.0%
5 (0.5% each)	*Corynebacterium*, *Limimaricola*, *Limnohabitans*, *Qipengyuania* and *Sulfitobacter* spp.	99.0–99.8%
2 (0.5% each)	*Allofrancisella* and *Lysobacter* spp.	93.2–97.2%
1 (0.5% each)	*Stutzerimonas* sp.	89.5%

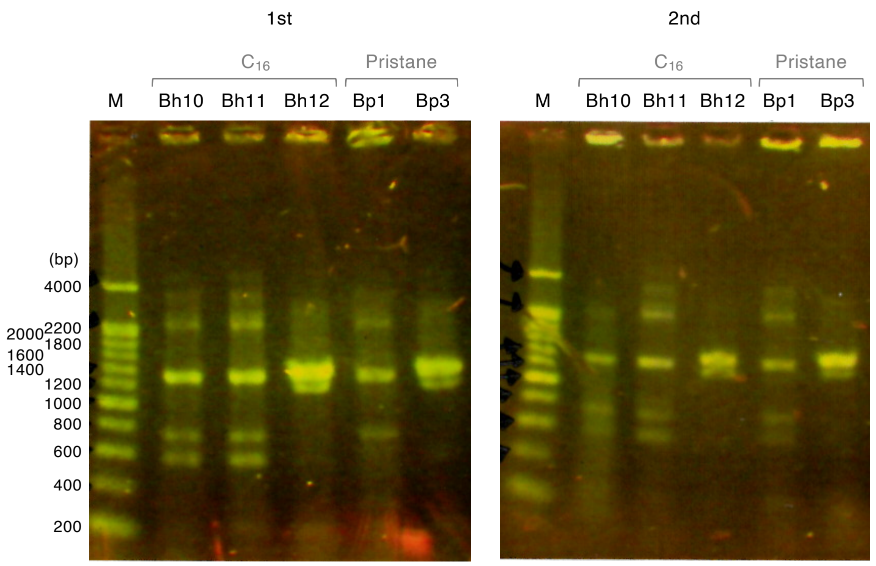

Fig. S1. Repetitive extragenic palindromic sequence PCR (rep-PCR) analysis for strain typing. Isolate Bh10 showed the same rep-PCR pattern as isolates Bh11 and Bp1, while isolate Bh12 showed the same pattern as isolate Bp3. Isolate Bh11 was obtained from the *n*-hexadecane (C_{16})-enriched culture (C_{16}) and degraded C_{16}, while isolates Bp1 and Bp3 were obtained from the pristane-enriched culture (pristane) and degraded pristane. Rep-PCR was performed twice for confirmation (1st and 2nd). M, marker.

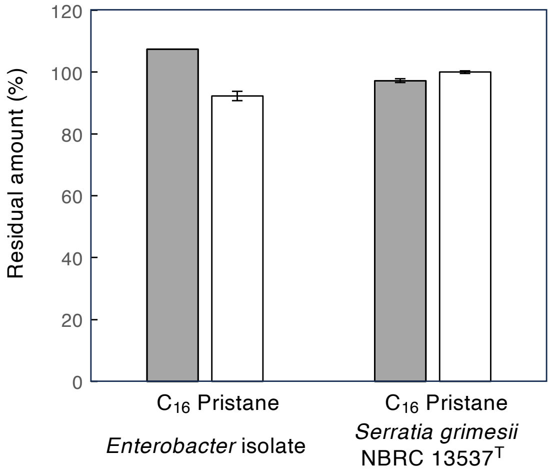

Fig. S2. Degradation of C_{16} or pristane (0.04%, vol/vol) in an inorganic medium, MP, by an *Enterobacter* isolate or *Serratia grimesii* NBRC 13537^T at 20 °C for 3 weeks. Data were obtained through GC-MS. Non-inoculated sterile samples were similarly incubated and served as controls (100%). These were normalized to equivalent *n*-tetracosane added just prior to extraction. Error bars represent standard errors.

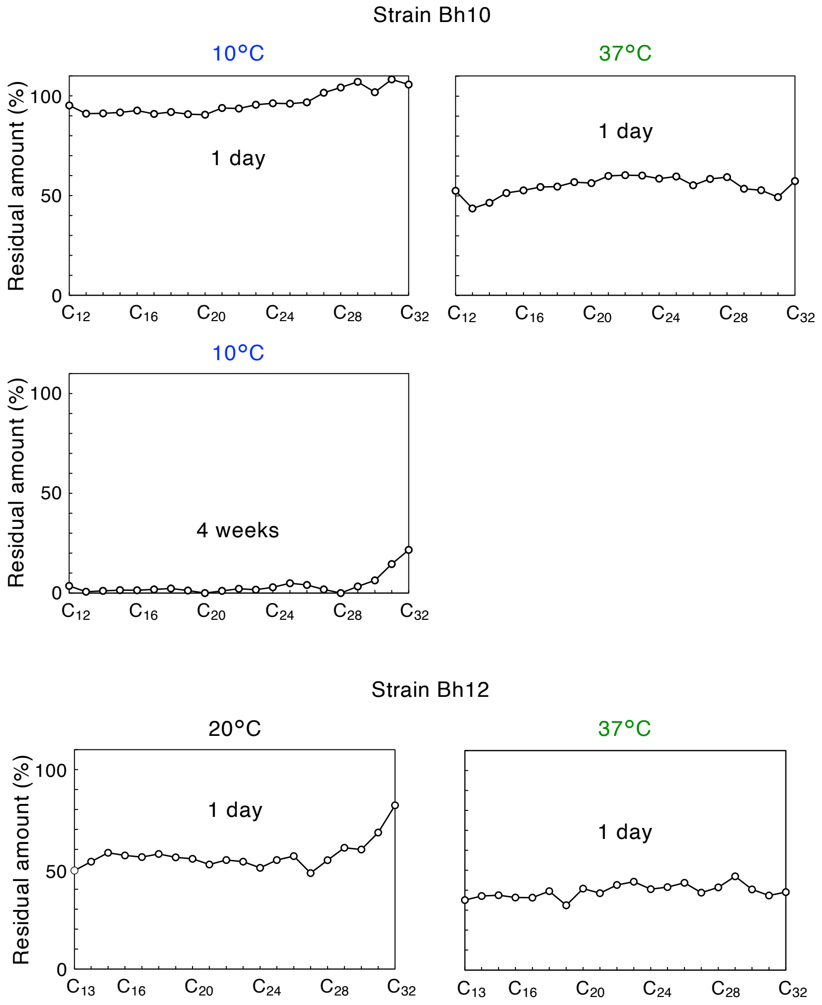

Fig. S3. Chain-length preference for *n*-alkanes, observed during the degradation of *n*-alkanes in crude oil (0.1%, vol/vol) in MP medium by isolate Bh10 or Bh12 (Fig. 3A). Data are shown as in Fig. 3B. The *n*-alkane degradation at 10 and 37 °C by isolate Bh10 and at 20 °C (preference for short *n*-alkanes) and 37 °C by isolate Bh12 are shown.

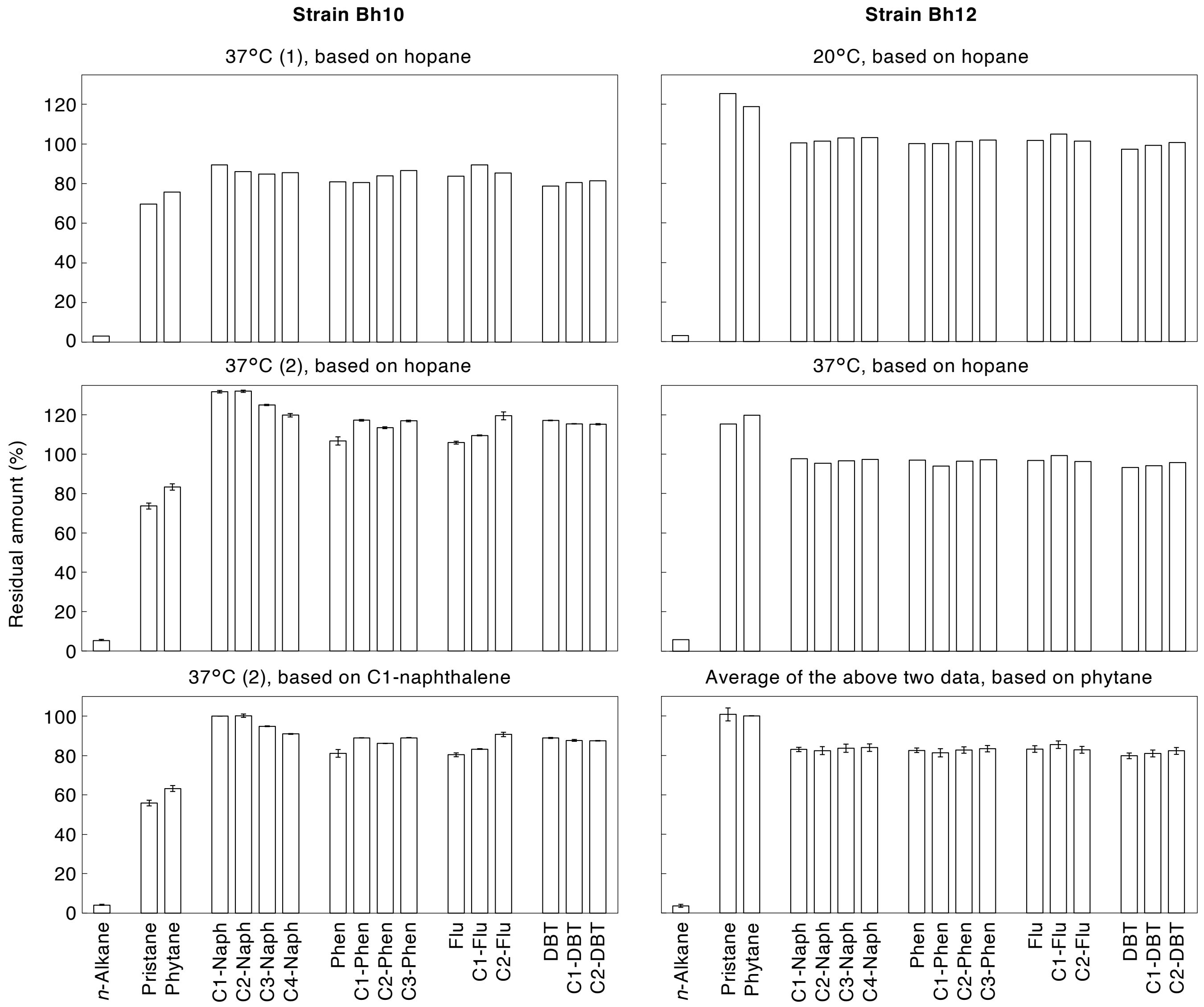

Fig. S4. Degradation of aromatics in crude oil (0.1%, vol/vol) in MP medium, observed at day 28 in Fig. 3, by isolate Bh10 or Bh12. Degradation of *n*-alkanes and branched alkanes (pristane and phytane) observed at the same time is also shown. Data were obtained through GC-MS. Non-inoculated sterile samples were similarly incubated and served as controls (100%). These were normalized to 17*α*(*H*),21*β*(*H*)-hopane contained in crude oil (upper and center). Using strain Bh10, hopane was suggested to be degraded more rapidly than aromatics at 37 °C (2) (center). Thus, the data at 37 °C (2) are also shown based on C1-naphthalene (bottom). The data at 37 °C (2) were from two samples prepared simultaneously. Difference in the degradation patterns between two experiments (37 °C (1) and 37 °C (2); upper and center) could be due to compositional change in crude oil during storage. Using strain Bh12, hopane was suggested to be degraded more rapidly than branched alkanes (upper and center). Thus, the averages of the two data are shown based on phytane (bottom). Error bars represent standard errors. C1-, methyl; C2-, dimethyl or ethyl; C3-, trimethyl, methyl ethyl, propyl, or isopropyl; Naph, Naphthalene; Phen, Phenanthrene; Flu, Fluorene; DBT, Dibenzothiophene.

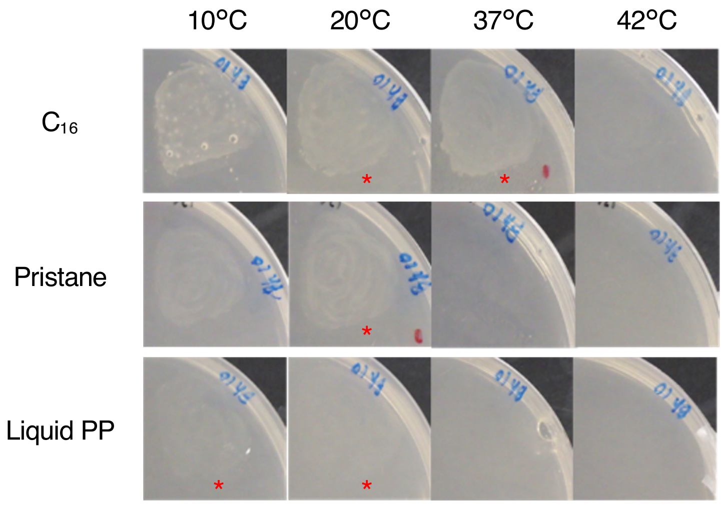

Fig. S5. Optimum temperature for growth on C_{16}, pristane or liquid PP by isolate Bh10. Isolate Bh10 pregrown on an LB plate was plated on MP plates covered with 3 µl C_{16}, pristane or liquid PP and incubated at 10, 20, 37 or 42 °C for 14 days (with pristane) or 18 days (with C_{16} and liquid PP). During incubations, isolate Bh10 grew fastest (*) at 20–37 °C on C_{16}, 20 °C on pristane, and 10–20 °C on liquid PP.

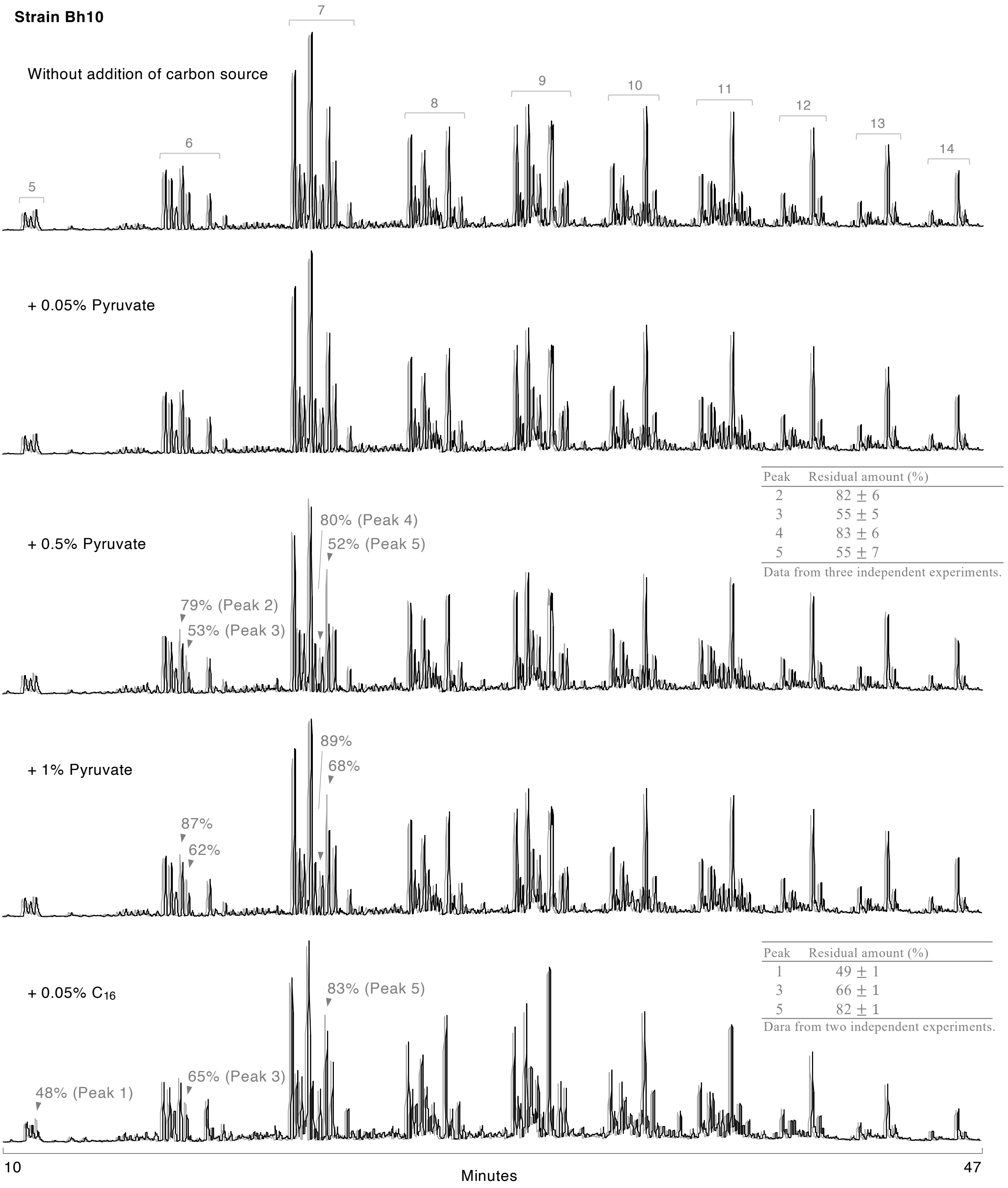

Peak	Residual amount (%)
2	82 ± 6
3	55 ± 5
4	83 ± 6
5	55 ± 7

Data from three independent experiments.

Peak	Residual amount (%)
1	49 ± 1
3	66 ± 1
5	82 ± 1

Dara from two independent experiments.

Fig. S6. Degradation of liquid PP (0.04 %, vol/vol) in MP medium by isolate Bh10 with or without C_{16} or pyruvate at 20°C for 4 weeks. GC-MS total ion chromatograms are shown. Concentrations of C_{16} (vol/vol) or pyruvate (wt/vol) are indicated. PP pentamers to tetradecamers are indicated as numbers above the peaks. Liquid PP in non-inoculated control samples is shown superimposed in gray and at a slightly left position. These are normalized to equivalent *n*-dodecane added just prior to extraction. Degraded peaks and the residual amounts are indicated. In the inset tables, each value is the mean ± standard error from two or three independent experiments.

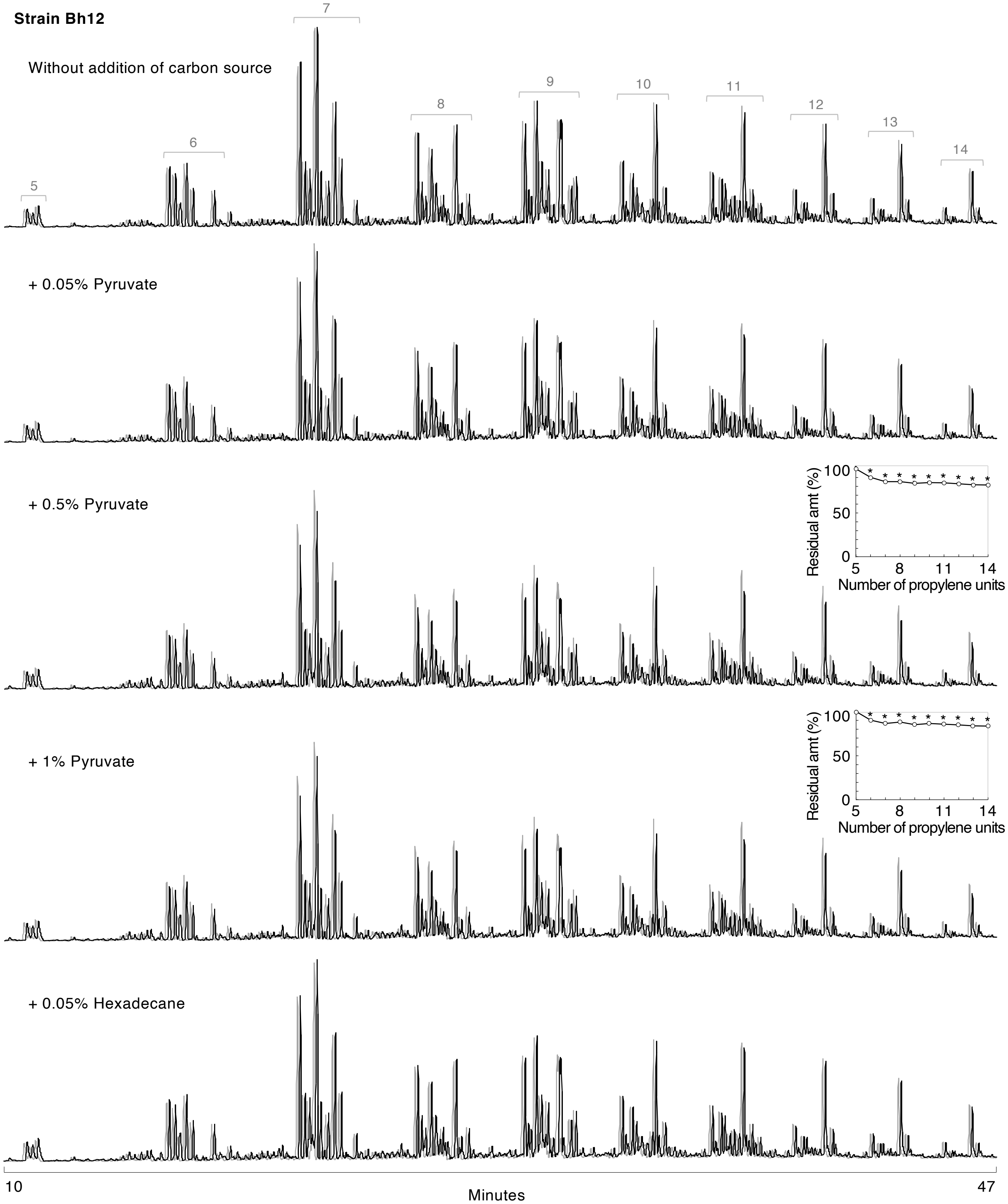

Fig. S7. Degradation of liquid PP (0.04 %, vol/vol) in MP medium by isolate Bh12 with or without C_{16} or pyruvate at 20°C for 4 weeks. GC-MS total ion chromatograms are shown. Concentrations of C_{16} (vol/vol) or pyruvate (wt/vol) are indicated. PP pentamers to tetradecamers are indicated as numbers above the peaks. Liquid PP in non-inoculated control samples is shown superimposed in gray and at a slightly left position. These are normalized to equivalent *n*-dodecane added just prior to extraction. When degradation was observed, the residual amount (amt) of each PP oligomer, using the main peak(s), is also shown as an inset. Error bars, representing standard errors from these main peaks of each oligomer, were smaller than the size of the symbols. Student's *t*-test of the two data with 0.5 and 1% pyruvate showed that the residual amounts of PP hexamers to tetradecamers were significantly lower (*) than the amounts of each PP oligomer in the non-inoculated control samples (100%) (each p value was <0.05).

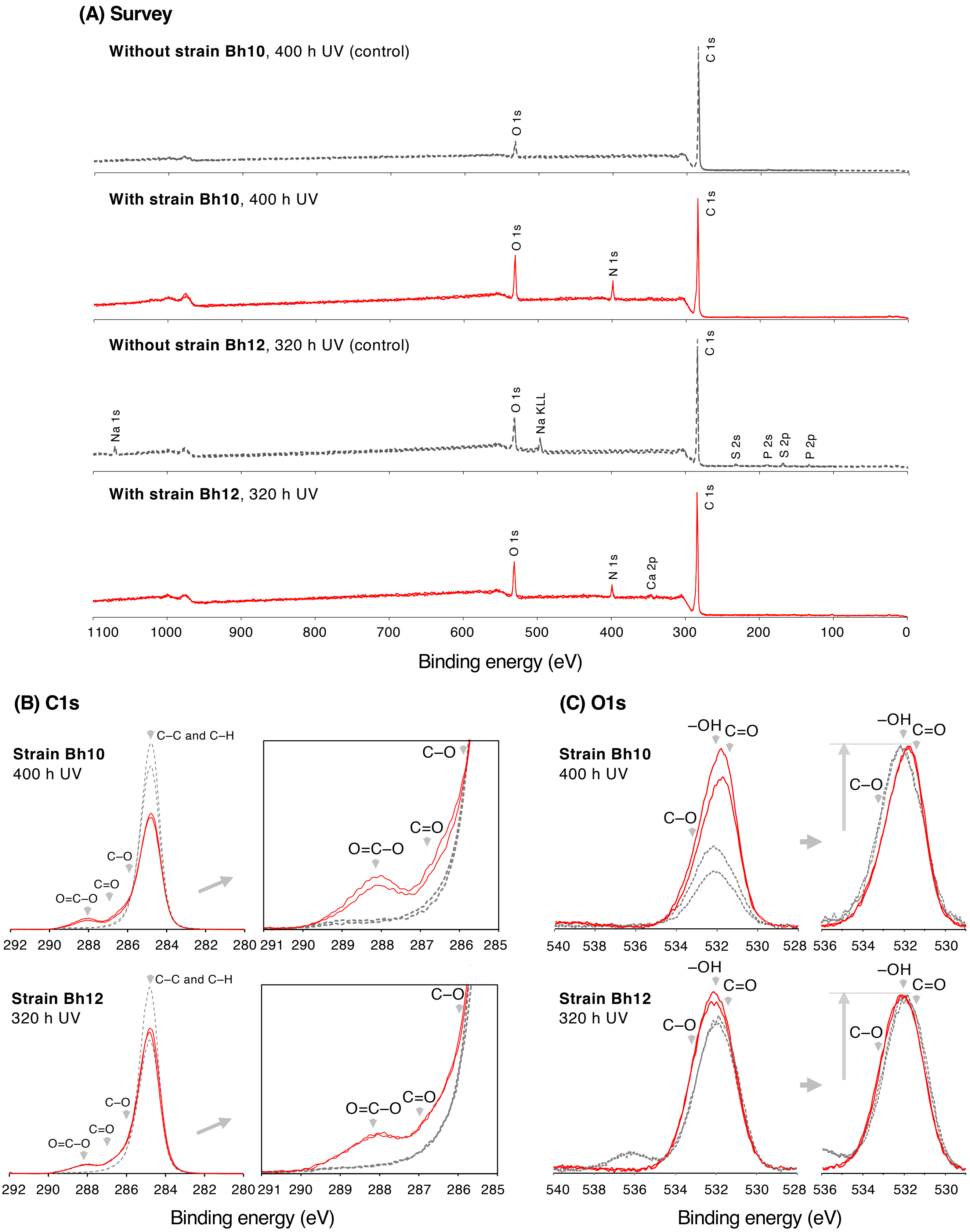

Fig. S8. XPS spectra of (A) survey, (B) C1s, and (C) O1s for PE film incubated in MP medium at 37 °C with strain Bh10 or Bh12 and 0.021% (vol/vol) C_{16} for 4 weeks. The spectra for PE film incubated in the same way without strains (controls) are shown as dashed lines. PE film irradiated with UV for 320 or 400 h was used. Two points were measured on each film. In C1s spectra, figures on the right are expansions of the figures on the left. In O1s spectra, figures on the right are normalized by the peak intensities. Arrow heads indicate the peaks of resolved spectra (in C1s; Fig. S9) or presumed resolved spectra (in O1s) for the linkages.

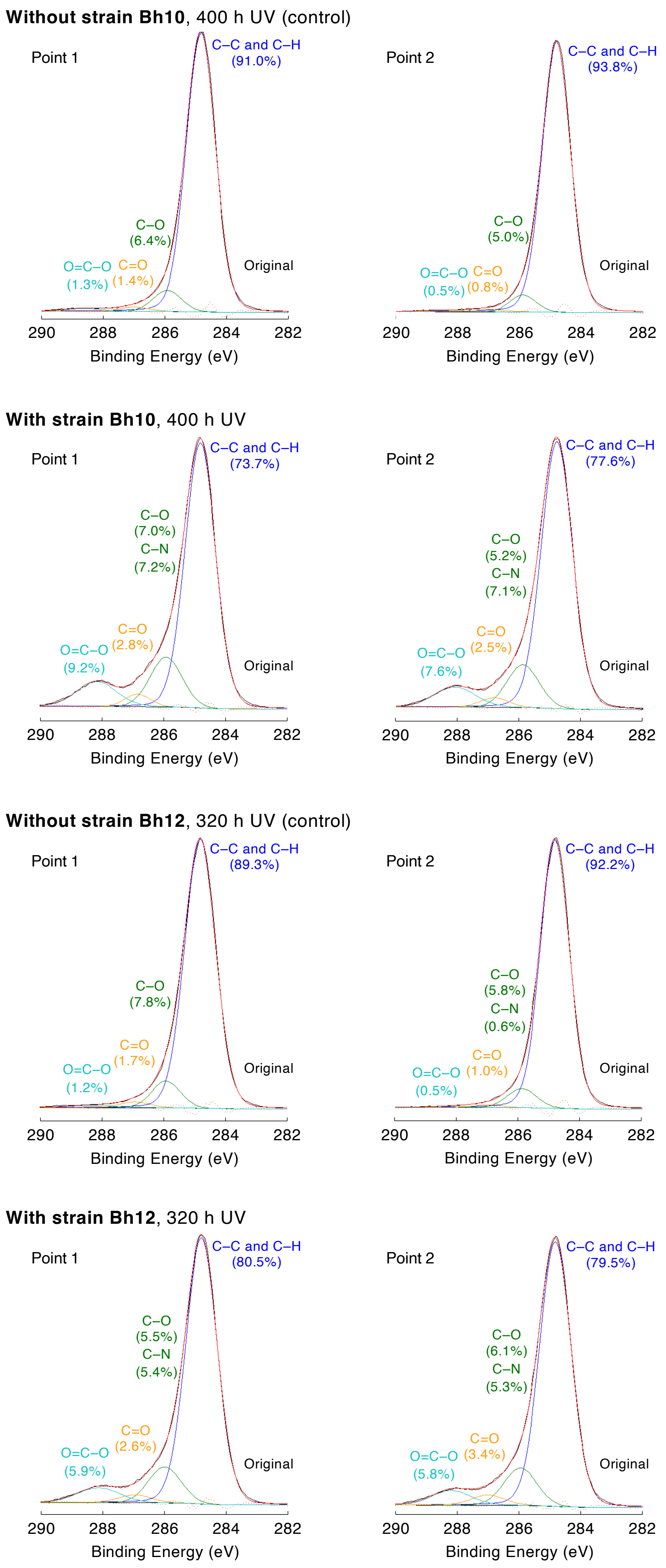

Fig. S9. Deconvoluted C1s XPS spectra in Fig. S8. Ratios of the peak areas are also given.

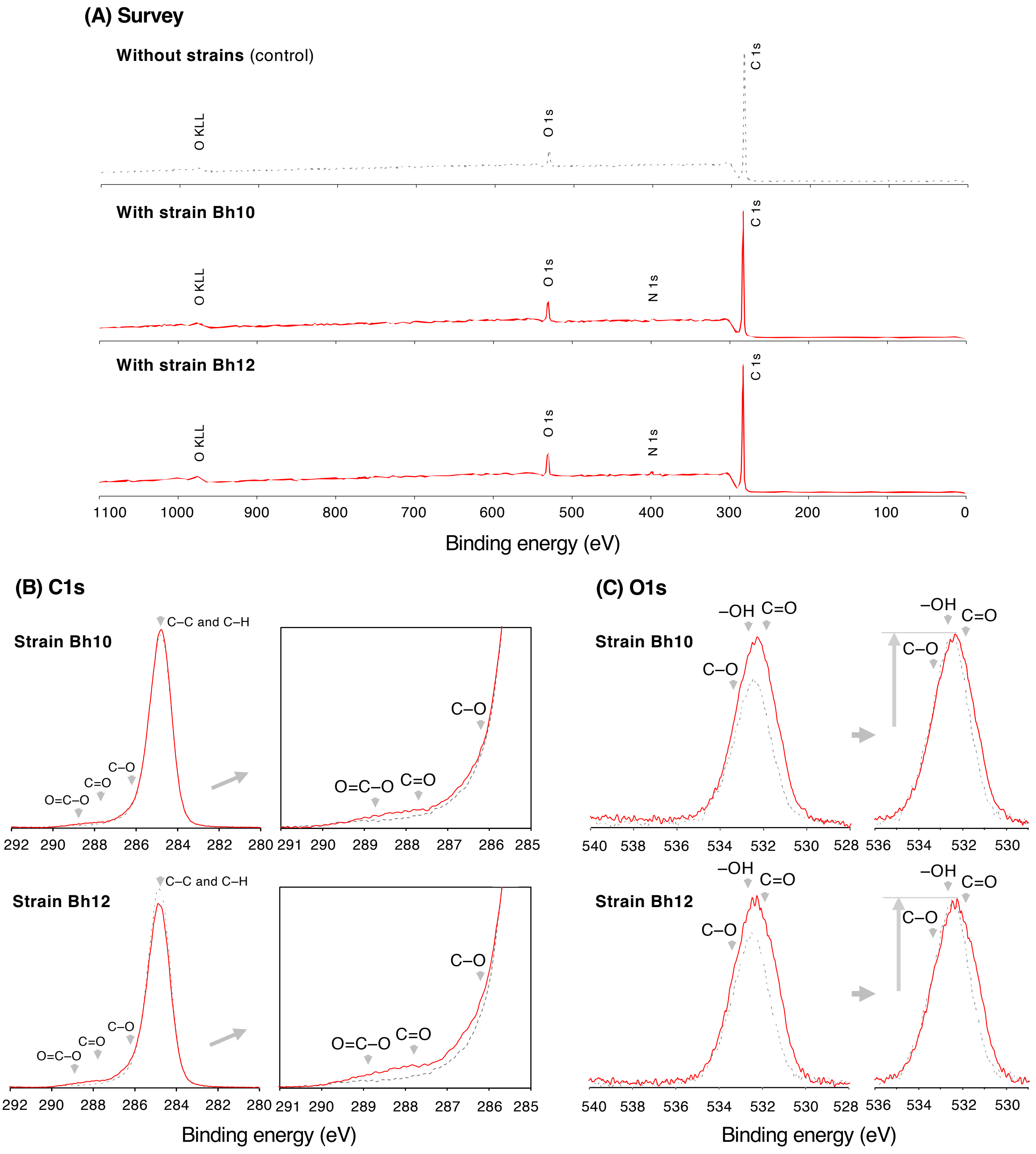

Fig. S10. XPS spectra of (A) survey, (B) C1s, and (C) O1s for PP film incubated in MP medium at 20 °C with strain Bh10 or Bh12 and 0.5% (wt/vol) pyruvate for 4 weeks. The spectra for PP film incubated in the same way without strains (controls) are shown as dashed lines. The spectra are for the films irradiated with UV for 75 h. In C1s spectra, figures on the right are expansions of the figures on the left. In O1s spectra, figures on the right are normalized by the peak intensities. Arrow heads indicate the peaks of resolved spectra (in C1s; Fig. S11) or presumed resolved spectra (in O1s) for the linkages. Areas (500 μm × 300 μm) were measured.

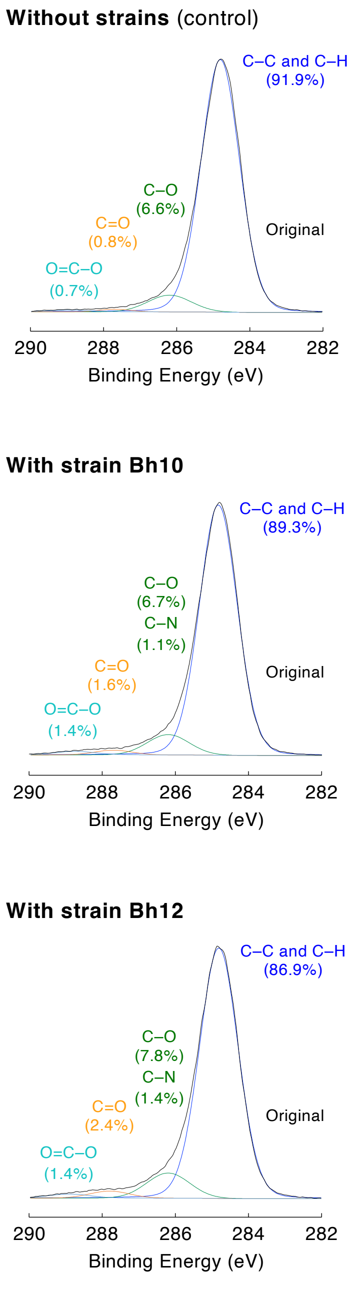

Fig. S11. Deconvoluted C1s XPS spectra in Fig. S10. Ratios of the peak areas are also given.
